# A DFT study of superior adsorbate–surface bonding at Pt-WSe_2_ vertically aligned heterostructures upon NO_2_, SO_2_, CO_2_, and H_2_ interactions

**DOI:** 10.1038/s41598-024-65213-y

**Published:** 2024-07-08

**Authors:** Aditya Kushwaha, Neeraj Goel

**Affiliations:** Department of Electronics and Communication Engineering, Netaji Subhas University of Technology, Dwarka, New Delhi 110078 India

**Keywords:** Pt-WSe_2_, Adsorption, 2D Material, Gas sensor, Sensitivity, Density functional theory, Engineering, Materials science, Nanoscience and technology

## Abstract

This study investigates the potential of platinum (Pt) decorated single-layer WSe_2_ (Pt-WSe_2_) monolayers as high-performance gas sensors for NO_2_, CO_2_, SO_2_, and H_2_ using first-principles calculations. We quantify the impact of Pt placement (basal plane vs. vertical edge) on WSe_2_’s electronic properties, focusing on changes in bandgap (ΔE_g_). Pt decoration significantly alters the bandgap, with vertical edge sites (T_V-WSe2_) exhibiting a drastic reduction (0.062 eV) compared to pristine WSe_2_ and basal plane decorated structures (T_BH_: 0.720 eV, T_BM_: 1.237 eV). This substantial ΔE_g_ reduction in T_V-WSe2_ suggests a potential enhancement in sensor response. Furthermore, T_V-WSe2_ displays the strongest binding capacity for all target gases due to a Pt-induced “spillover effect” that elongates adsorbed molecules. Specifically, T_V-WSe2_ exhibits adsorption energies of − 0.5243 eV (NO_2_), − 0.5777 eV (CO_2_), − 0.8391 eV (SO_2_), and − 0.1261 eV (H_2_), indicating its enhanced sensitivity. Notably, H_2_ adsorption on T_V-WSe2_ shows the highest conductivity modulation, suggesting exceptional H_2_ sensing capabilities. These findings demonstrate that Pt decoration, particularly along WSe_2_ vertical edges, significantly enhances gas sensing performance. This paves the way for Pt-WSe_2_ monolayers as highly selective and sensitive gas sensors for various applications, including environmental monitoring, leak detection, and breath analysis.

## Introduction

Gas sensors play a crucial role across various domains, contributing to air quality, climate change mitigation, industrial safety, and medical diagnostics^1,2^. The development of sensors for NO_2_^3,4^, CO_2_^5,6^, SO_2_^7,8^, and H_2_^9,10^ is imperative due to their significant impact on human health, the environment, and industrial processes. NO_2_, a major air pollutant, contributes to respiratory issues and environmental degradation^11^. CO_2_ levels serve as critical indicators of indoor air quality and are linked to climate change^12^. SO_2_, an industrial byproduct, is associated with air pollution and respiratory ailments^13^. H_2_, widely used in industrial processes, poses safety risks in certain concentrations^14^. Efficient gas sensors for these compounds are essential for real-time monitoring, early hazard detection, and proactive measures to ensure environmental sustainability and public health.

Highly sensitive and selective sensors are crucial for the swift and accurate detection of hazardous gases to minimize risks. Various sensors have been developed to address these challenges. Conventional gas sensors, relying on metal oxides and polymers, face limitations such as reduced selectivity, high operating temperatures, extended response and recovery times, limited sensitivity to low concentrations, and vulnerability to environmental conditions^15,16^. Following the success of utilizing graphene as a gas sensor^17,18^, researchers have shifted their focus to exploring two-dimensional (2D) transition metal dichalcogenides (TMDs) materials^19,20^. These materials, with exceptional structural configurations, offer large surface-to-volume ratios and the ability to alter their electronic properties^21,22^. The interest in 2D materials is driven by their potential to enhance gas sensing capabilities, enabling more accurate and selective detection of various gases.

Recent reports highlight tungsten diselenide (WSe_2_) as a superior gas sensor material among TMDs. WSe_2_ exhibits enhanced sensitivity, tunable bandgap for specific gas detection, and efficient charge transport, providing swift and precise responses^23,24^. Its larger surface area and chemical stability contribute to improved adsorption and durability, while low noise levels and selective customization enhance accuracy. WSe_2_’s compatibility with microfabrication enables compact devices and effective room-temperature operation. Ongoing research, exemplified by Guo et al.^25^, showcases WSe_2_ nanosheets with high sensitivity to NO_2_ at room temperature, high selectivity, and stability for 8 weeks. Similarly, Wu et al.^26^ demonstrate a WSe_2_ monolayer-based NO_2_ gas sensor with detection capabilities across varying concentrations and temperatures, emphasizing rapid recovery, high selectivity, reversibility, and stability over 60 days for NO_2_ detection.

The use of 2D materials, especially TMDs like WSe_2_, in gas sensors is gaining attention for their superior performance attributed to a high surface-to-volume ratio^27^. Monolayers of WSe_2_, a significant member of dichalcogenides, exhibit remarkable electronic properties and substantial surface area, enhancing the sensitivity and range of gas sensors with specific doping or surface modifications. This is crucial for devices detecting harmful gases (NO_2_, HCHO, NH_3_, SF_6_ decomposed gases) in residential and occupational environments^28^. Noble metal-decorated WSe_2_ is recognized as a promising material for gas sensing applications. Pt atoms are a frequent choice for absorbing molecules on TMDs due to several key properties. Pt’s well-known catalytic activity aids in weakening bonds and facilitating desired chemical reactions on the TMD surface. It can also modify the TMD’s work function, influencing the adsorption and release of specific gas molecules for targeted sensing or manipulation. Additionally, Pt offers good thermal and chemical stability, making it practical for applications where the TMD-Pt composite might encounter harsh environments. Compared to generic metal decoration, Pt provides a more targeted approach due to its specific catalytic properties and work function effects. Furthermore, Pt can influence the TMD’s electronic structure at the interface, potentially enhancing its conductivity. The combination of Pt and the TMD can even create synergistic effects, leading to superior performance compared to either material alone. Density Functional Theory (DFT) computations play a crucial role in examining gas sensing and functionalization of single-atom catalysts, revealing improved sensitivity of Pd-functionalized WSe_2_ systems in detecting harmful gases. Doping WSe_2_ with noble metals (Pd, Ag, Au, Pt) enhances gas sensing, with Ag-WSe_2_ showing potential for efficient NO_2_ sensing^29^. Noble metal catalysts like Pt/Pd are proposed to boost H_2_ gas sensor sensitivity at room temperature. Studies indicate that Pd film on TiO_2_ enhances H_2_ sensitivity^30^, while Pt nanoparticles in F-MWCNT/TiO_2_/Pt hybrid achieve notable sensitivity to H_2_ molecules at specific concentrations^31^.

Comprehending the importance of further theoretical and experimental exploration in understanding and optimizing the gas sensing capabilities of single-atom Pt-decorated WSe_2_ (Pt-WSe_2_). While significant progress has been made in comprehending the effects of single-atom doping, especially in materials like molybdenum disulfide (MoS_2_), there remains a notable gap in theoretical inquiries regarding the adsorption phenomena of representative gases (H_2_, NO_2_, CO_2_, and SO_2_) on monolayers of Pt-WSe_2_. The research aims to address this gap by investigating the interactions between these target gas molecules and WSe_2_ layers coated with single-atom Pt, providing valuable insights for gas sensing applications. Consequently, this methodology integrates the distinctive characteristics of WSe_2_, including its elevated surface-to-volume ratio and semiconducting nature, with the catalytic attributes of Pt, leading to an enhanced gas sensing platform with improved efficiency. Gas sensing has advanced significantly as a result of the combination of these materials and methods.

This study employs DFT to investigate the gas sensing capabilities of a Pt-WSe_2_-based system toward specific target gases (H_2_, NO_2_, CO_2_, and SO_2_). Pt-atom is strategically decorated over the basal and vertical edges of the WSe_2_ monolayer. The analysis encompasses electronic characteristics, including band structure, density of states (DOS), charge density difference (CDD), and population analysis for different gases. The vertical configuration of Pt-WSe_2_ demonstrates outstanding sensitivity and recovery time for H_2_, attributed to the spillover effect. The findings propose a promising strategy to enhance the sensing response to hydrogen gas, elucidating the impact of Pt functionalization on the sensing mechanism.

## Computational methods

This paper employs the Dmol^3^ package in Material Studio software for all theoretical and DFT calculations^32,33^. The Perdew–Burke–Ernzerhof (GGA-PBE) generalized approximation is utilized to handle electron exchange correlation accurately^34,35^. The study incorporates Density Functional Theory with dispersion (DFT-D) and van der Waals (vdWs) interactions to enhance accuracy, addressing the traditional DFT’s challenges in capturing weak forces^36,37^. Tkatchenko and Scheffler’s (TS) method is applied for precise simulation of vdWs interactions^38,39^. DFT semi-core pseudopotentials (DSPP) replace core electrons of specific atoms, enhancing accuracy in electronic interaction descriptions^40^. DSPP, combined with Double numerical plus polarization (DNP) basis sets, is used to describe electronic wavefunctions, improving predictions of molecular properties and behaviour. The approach involves two sets of functions for core and valence electrons, including extra functions for electron density changes due to neighbouring atoms (polarization). This comprehensive methodology enhances accuracy in describing electronic interactions and predictions of molecular behaviour. In geometry optimizations, the criteria for energy convergence, maximum force, and maximum displacement were set at 1.0 × 10^–5^ Ha, 0.002 Ha/Å, and 0.005 Å, respectively. A global orbital cutoff radius of 5.0 Angstroms was used. For calculating DOS, a 7 × 7 × 1 Monkhorst–Pack k-point grid was employed, while a 3 × 3 × 1 Monkhorst–Pack k-point grid was utilized for other calculations, ensuring an accurate representation of the Brillouin zone^41^. The WSe_2_ structure was cleaved along the [1 0 0] direction to expose the vertical edge. Moreover, for the analysis, we have considered a 3 × 3 × 1 WSe_2_ supercell with a vacuum region of c = 15 Å in order to avoid contact with surrounding WSe_2_ layers. Furthermore, the study involves calculating adsorption energy (*E*_*ad*_) using Eq. (1), respectively. The CDD for the adsorption configuration (*Δρ*) is determined using Eq. (2). (Refer to Eqs. (1), (2) in the supporting information). Moreover, in physisorption, the dominant interactions between the adsorbate and adsorbent are typically weak, characterized by van der Waals forces or dipole–dipole interactions. This is reflected in the observation that the sum of covalent radii tends to exceed the adsorption distance. Conversely, chemisorption involves stronger interactions, often leading to the formation of chemical bonds between the adsorbate and adsorbent surface. Here, the adsorption distance closely aligns with the sum of covalent radii, indicating the potential for chemical bond formation. This interpretation is substantiated by previous studies utilizing density functional theory (DFT) calculations, which have consistently demonstrated this correlation^42,43^.

## Results and discussion

**Figure 1:**
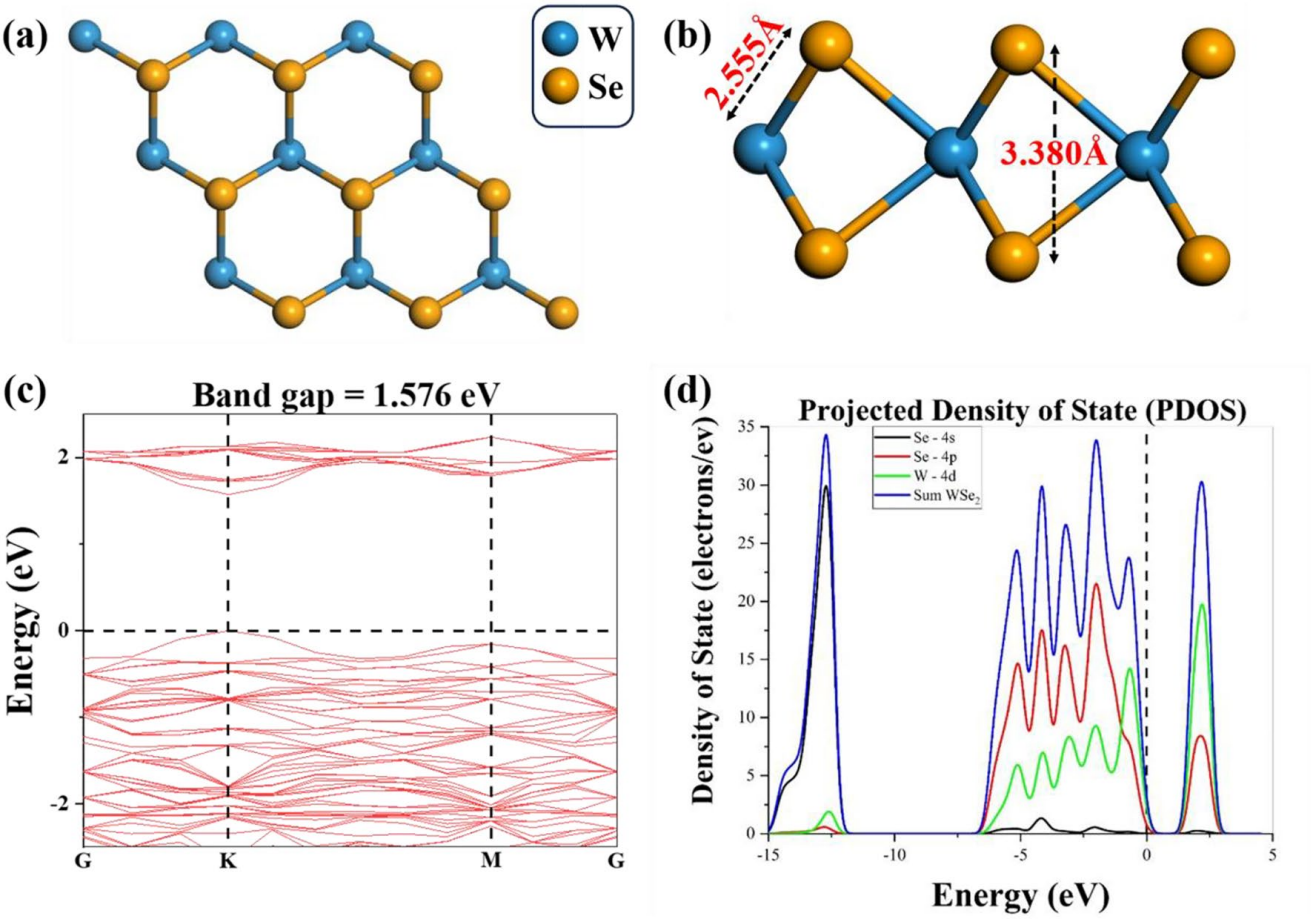
(**a**) Top surface, and (**b**) side profile, of the 3 × 3 WSe_2_ monolayer. (**c**) The electronic band, and (**d**) the projected density of states (PDOS) structure of the system is presented, herein the horizontal and vertical dashed line signifies the Fermi level in the band structure and PDOS, respectively.

To examine the gas sensing behaviour of WSe_2_, firstly we examine the physical and electrical properties of individual monolayer structures of WSe_2_ as shown in Fig. 1a, focusing on its geometric structure. The available chalcogen vacancies play a significant role in the electronic configuration of TMDs^44^. Subsequently, we investigate the properties of Pt-coated WSe_2_. In this study, we specifically consider WSe_2_ with a monolayer structure, where cutting the (001) plane results in the formation of hexagonal lattices with a honeycomb-like arrangement. Following structural optimization, the monolayers of WSe_2_ displayed a bond length of 2.555 Å between W and Se atoms, and a bond length of 3.380 Å between the Se atoms (Fig. 1b). The calculated outcomes and the reported values of 2.54 Å (W-Se) exhibited a notable concordance, indicating a satisfactory agreement between them^45^. Moreover, in Fig. 1c we showed the bandgap for the WSe_2_ monolayer which shows the value of 1.576 eV between the conduction and valence band representing good agreement with previously reported work^46^. Figure 1d presents the projected density of states (PDOS) for the pristine WSe_2_ monolayer. The PDOS reveals significant hybridization between the W 4d orbitals and the Se 4s and 4p orbitals, indicating the formation of covalent bonds between the W and Se atoms.

**Figure 2:**
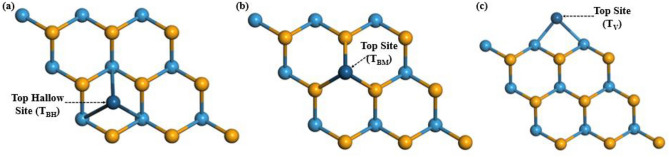
Three possible configurations for decorating a WSe_2_ monolayer with platinum atom: (**a**) at the hollow hexagonal site of the WSe_2_ basal plane (T_BH-WSe2_), (**b**) atop a W atom within the basal plane (T_BM-WSe2_), and (**c**) at the vertical edge of the WSe_2_ monolayer (T_V-WSe2_).

This study employs computational simulations to investigate the impact of Pt decoration on the electronic properties of a WSe_2_ monolayer. Moreover, we considered three potential sites for decoration and they are labelled as T_BH-WSe2_ (Pt-atom decorated over the hallow hexagonal site in WSe_2_ basal plane configuration), T_BM-WSe2_ (Pt-atom decorated over the W atom in WSe_2_ basal plane configuration), and T_V-WSe2_ (Pt-atom decorated at the vertical edge of WSe_2_) as illustrated in Fig. 2a–c.

Furthermore, the optimized configurations of T_BH-WSe2_, T_BM-WSe2_, and T_V-WSe2_ reveal bandgap and DOS values of 0.720 eV, 1.237 eV, and 0.062 eV, respectively (Figs. S1–S3). We have analyzed the electronic spin for Pt-WSe_2_ (Fig. S11(a1–a3) in Supplementary Information for details). Pt functionalization on the WSe_2_ monolayer induces the hybridization of electronic orbitals, creating new states within the bandgap and reducing bandgap values. The Pt placement significantly influences the electronic properties of WSe_2_, highlighting potential functionality tailoring. Adsorption energy is negative, indicating an exothermic process. Bond lengths vary slightly post-optimization, with bond lengths between Pt-atom and W-atom for T_BH-WSe2_ and T_V-WSe2_ at 3.917 Å and 2.802 Å, respectively, and Pt and Se atom bond lengths in T_BM-WSe2_ at 2.429 Å. Mulliken and Hirshfeld analyses show electron depletion at Pt, indicating positive charges (0.050 e, 0.056 e, 0.062 e) in T_BH-WSe2_, T_BM-WSe2_, and T_V-WSe2_, respectively (Fig. S4). Previous studies suggest that Pt-atom promotes catalytic oxidation, leading to hole accumulation layers. Rosy and green colours in Fig. S4 represent electron depletion and accumulation, respectively. The thermodynamic stability of T_BH-WSe2_, T_BM-WSe2_, and T_V-WSe2_ combinations was evaluated by molecular dynamics (MD) simulations performed at 500 K to examine their thermal stability. Moreover, phonon dispersion simulations were conducted to analyze the vibrational modes of T_V-WSe2_, as shown in Fig. S5. Additionally, the optimized arrangement of target gases is depicted in Fig. S6.

### H_2_ adsorption

**Figure 3:**
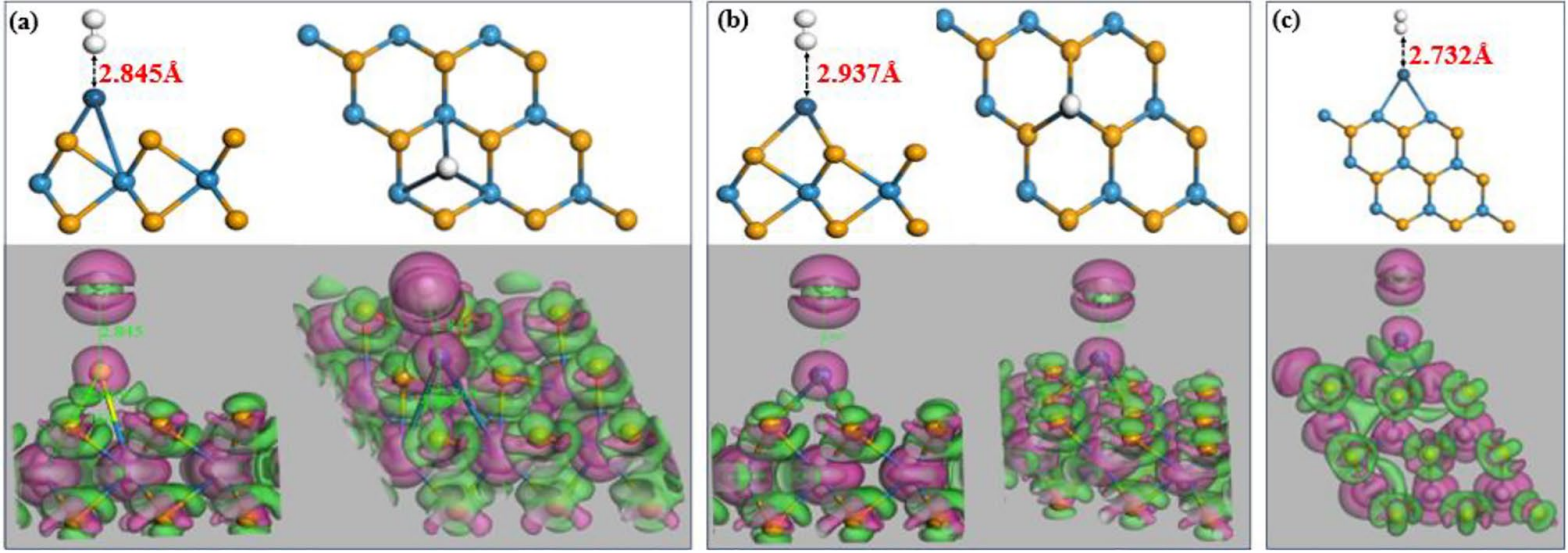
Most stable configuration and corresponding charge density distribution (CDD) following H_2_ adsorption at three different sites where a Pt is decorated: (**a**) the hollow site in the basal configuration, (**b**) above the W atom in the basal configuration, and (**c**) along the vertical edge of WSe_2_. In the CDD diagram, electron depletion manifests itself in a rosy colour, while electron accumulation is represented by a green colour.

Figure 3 depicts the stable adsorption and CDD of the H_2_ gas molecule for all three proposed structures namely T_BH-WSe2_ (Fig. 3a), T_BM-WSe2_ (Fig. 3b), and T_V-WSe2_ (Fig. 3c). It can be observed that there is elongation in the H–H bond (~ 0.212 Å) which denotes the dissociation of the H_2_ molecule and supports the spillover effect^47^ due to the decoration of Pt. The enhanced H_2_ detection capabilities of Pt-WSe_2_ arise primarily from two contributing factors: the presence of Pt-atom and the interfacial contact between Pt and WSe_2_. Pt-atom acts as a potential candidate for efficient H_2_ sensing, facilitating the “spillover effect” where captured H_2_ molecules migrate to the WSe_2_ surface for subsequent adsorption. Moreover, the contact between Pt and WSe_2_ promotes the generation of new adsorption sites on the WSe_2_ monolayer, further enhancing the overall H_2_ binding capacity. Therefore, both the Pt-atom and the synergistic Pt-WSe_2_ interface play crucial roles in the superior H_2_ detection performance of Pt-WSe_2_. In the Pt-WSe_2_ system, upon interaction with H_2_ gas molecules, an initial adhesion occurs between the catalytic Pt-atom and the gas molecules, leading to subsequent dissociation into single hydrogen atoms. This establishes the conditions necessary for a chemical interaction to occur between H atoms and the WSe_2_ material, hence promoting the process of H atom diffusion into the WSe_2_ structure. This causes chemisorption among all three proposed configurations. Moreover, a reduction in electrical resistance when H_2_ comes in contact with the Pt-WSe_2_ sensor. The observed decrease in resistance implies that H_2_ tends to transfer electrons to the WSe_2_ material. In other words, H_2_ is a reducing nature gas due to which it will donate electrons to the system which is confirmed with the help of Mulliken and Hirshfeld’s analysis and the gap between Pt and H atom shown in Table 1.

**Table 1:** Summary of electron distribution and H_2_ gas adsorption distance in three proposed configurations.

System	Total charge over Pt		Total charge over H_2_		Adsorption distance between Pt and H atom (in Å)
	Mulliken	Hirshfeld	Mulliken	Hirshfeld	
T_BH-WSe2_	− 0.059e	− 0.0721e	0.004e	0.040e	2.845
T_BM-WSe2_	− 0.028e	− 0.0605e	0.004e	0.035e	2.937
T_V-WSe2_	− 0.036e	− 0.0696e	0.010e	0.037e	2.732

Consequently, it can be observed from Table 1 total charge over the H_2_ molecule is positive which displays the depletion of electrons. And these electrons are transmitted to the Pt-atom which possesses a negative value which is also illustrated in the CDD diagram. Furthermore, we have provided the electronic structure analysis (i.e., band structure, DOS, and PDOS) after the adsorption of the H_2_ gas molecule in the supplementary information (Fig. S7). We investigated the electronic spin response upon H_2_ adsorption (Fig. S11(b1–b3); refer to Supplementary Information for details). In addition, we have calculated the adsorption energies for the T_BH-WSe2_, T_BM-WSe2_, and T_V-WSe2_ configurations towards the H_2_ gas molecule and obtained the values of − 0.0293 eV, − 0.0788 eV, and − 0.1261 eV, respectively. It could be easily seen that the most favourable case for strong H_2_ adsorption was exhibited by T_V-WSe2_.

### NO_2_ adsorption

**Figure 4:**
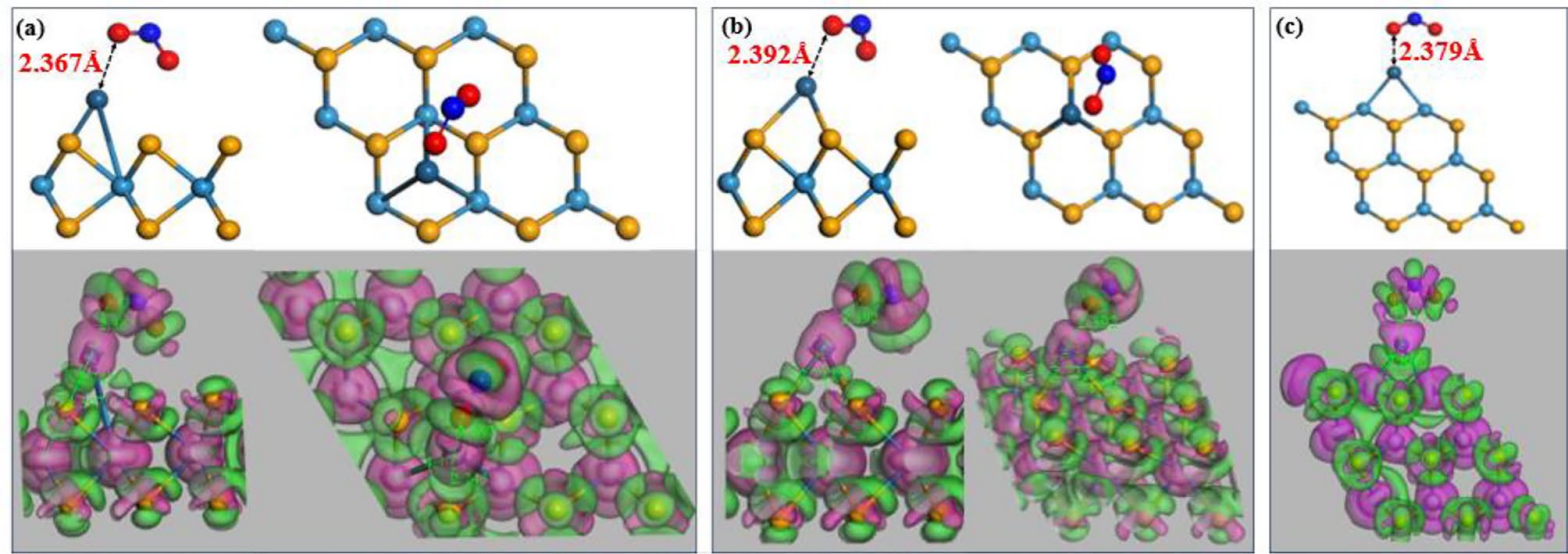
Optimal arrangement and associated charge density distribution after NO_2_ adsorption at three distinct locations where a Pt is functionalized: (**a**) the hollow site in the basal configuration, (**b**) positioned above the W atom in the basal configuration, and (**c**) situated along the vertical edge of WSe_2_. Electron depletion and accumulation are visualized through the application of rosy and green colours.

The Fig. 4 illustrates the stable adsorption and CDD of the NO_2_ gas molecule on three suggested structures: T_BH-WSe2_ (Fig. 4a), T_BM-WSe2_ (Fig. 4b), and T_V-WSe2_ (Fig. 4c). Notably, there is an elongation observed in the N–O bond, approximately to 0.060 Å, indicating the weak interaction between the Pt-atom and the NO_2_ molecule. Furthermore, this minor extension in bond is corroborated by the low binding capacity arising from the Pt decoration.

The Pt–N minimum adsorption distance, measuring 2.367 Å, surpasses the sum of relevant covalent radii (2.010 Å for Pt–N^48^), indicating a physisorption nature. It is evident that physisorption was consistently observed for NO_2_ in all three suggested arrangements. Additionally, it is essential to note that NO_2_ gas exhibits an oxidizing nature, causing it to withdraw electrons from the system. This aspect has been confirmed through the comprehensive analysis performed using Mulliken and Hirshfeld’s population analysis method, and the findings are presented in Table 2 with the adsorption length between Pt and O atom.

**Table 2:** Summarizing electron distribution and adsorption distances post-NO_2_ gas adsorption across three distinct proposed configurations.

System	Total charge over Pt		Total charge over NO_2_		Adsorption distance between Pt and O atom (in Å)
	Mulliken	Hirshfeld	Mulliken	Hirshfeld	
T_BH-WSe2_	0.073e	0.1553e	− 0.254e	− 0.2364e	2.367
T_BM-WSe2_	0.085e	0.1436e	− 0.25e	− 0.235e	2.392
T_V-WSe2_	0.103e	0.1389e	− 0.244e	− 0.2243e	2.379

As a result, Table 2 reveals that the total charge on the NO_2_ molecule is negative, signifying the accumulation of electrons within the molecule. These electrons are then extracted from the Pt-atom, which, as shown in the CDD diagram, possesses a negative value. Additionally, electronic structure analysis, including band structure, DOS, and PDOS after NO_2_ gas molecule adsorption, is available in the supplementary information (Fig. S8). To understand the interaction between the material and NO_2_ on a deeper level, we analyzed the electronic spin after gas adsorption as shown in Fig. S11(c1–c3) (see Supplementary Information for details). Additionally, we have conducted calculations for the adsorption energy of the T_BH-WSe2_, T_BM-WSe2_, and T_V-WSe2_ configurations upon exposure to NO_2_ gas molecules. The obtained values are − 0.4227 eV, − 0.3879 eV, and − 0.5243 eV, respectively. Notably, the most favourable scenario for moderate adsorption is observed in the case of T_V-WSe2_, signifying enhanced physisorption. The stronger adsorption energy of the T_V-WSe2_ system among all the proposed structures confirms more available W sites which enhance the adsorption towards the NO_2_ gas molecule^49^. Overall, in this case, the Pt-atom facilitates the weak vdW interaction force for the NO_2_ gas molecule.

### CO_2_ adsorption

**Figure 5:**
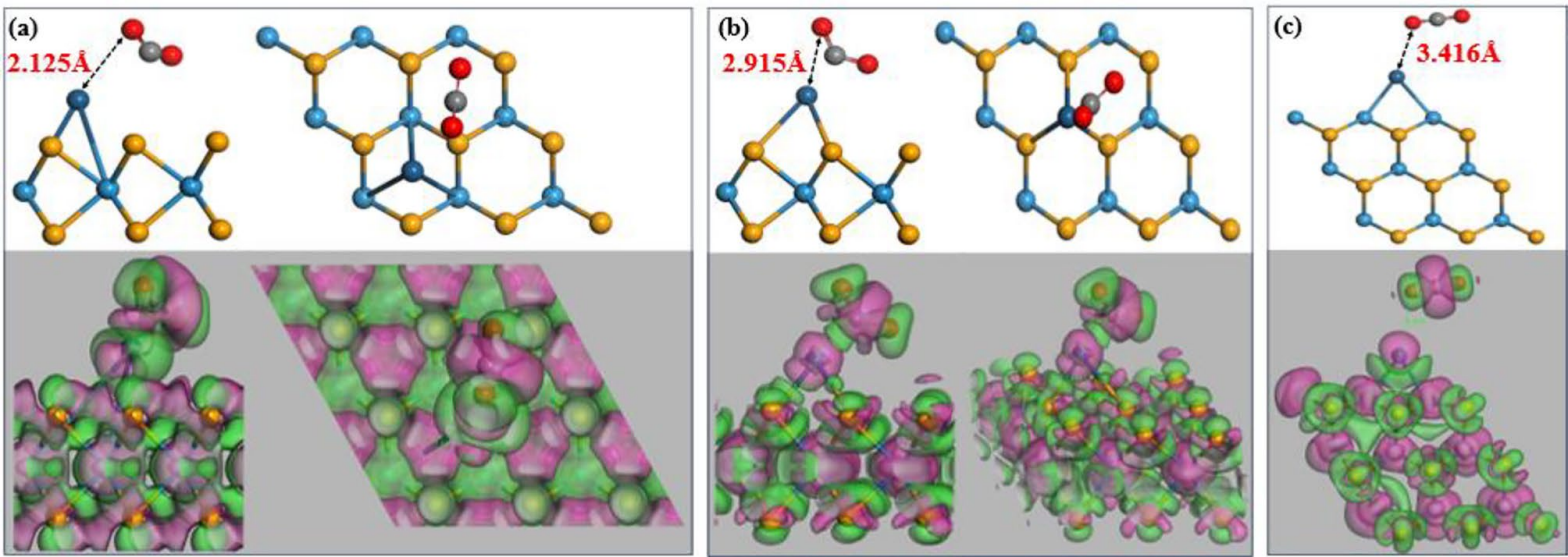
Most stable configuration and corresponding charge density distribution following CO_2_ adsorption at three different sites where a Pt is decorated: (**a**) the hollow site in the basal configuration, (**b**) above the W atom in the basal configuration, and (**c**) along the vertical edge of WSe_2_. Electron depletion and electron accumulation are mapped onto rosy and green colour regions, respectively.

In Fig. 5, we can observe the stable adsorption and dissociation of the CO_2_ gas molecule on three suggested structures: T_BH-WSe2_ (Fig. 5a), T_BM-WSe2_ (Fig. 5b), and T_V-WSe2_ (Fig. 5c). Significantly, the C–O bond exhibits an alteration of approximately 0.074 Å, indicating a weak vdW interaction between CO_2_ and Pt. Additionally, noticeable deformations are observed in the CO_2_ structure when it interacts with T_BH-WSe2_ and T_BM-WSe2_. This deformation is a result of an enhanced adsorption energy (~ − 0.50 eV) and less adsorption distance in comparison to the T_V-WSe2_-based configuration. In T_BH-WSe2_ and T_BM-WSe2_, the Pt-atom interacts with the C atom, exhibiting adsorption distances of 2.012 Å and 2.138 Å, respectively. For T_BH-WSe2_ and T_BM-WSe2_ the adsorption distance is below and above the sum of relevant covalent radii (2.050 Å for Pt and C^48^), indicative of chemisorption and physisorption, respectively. Conversely, in T_V-WSe2_, the Pt-atom interacts with the O atom, with an adsorption distance of 3.416 Å exceeding the sum of relevant covalent radii (2.020 Å for Pt and O^48^), suggesting a physisorption mechanism. These findings, supported by the CDD diagram (Fig. 5), elucidate distinct adsorption behaviours in the investigated Pt-WSe_2_ variants. Consequently, based on these observations, it can be concluded that chemisorption takes place in the case of T_BH-WSe2_, while physisorption occurs in the case of T_BM-WSe2_ and T_V-WSe2_ structures. Moreover, it is crucial to highlight that the CO_2_ gas demonstrates an oxidizing characteristic, thereby extracting electrons from the system. To confirm this aspect, we conducted a comprehensive analysis using Mulliken and Hirshfeld’s population analysis method, and the adsorption length is tabulated in Table 3.

**Table 3:** CO_2_ adsorption behaviour is presented through electron distribution and distance analysis in three configurations.

System	Total charge over Pt		Total charge over CO_2_		Adsorption distance between Pt and O atom (in Å)
	Mulliken	Hirshfeld	Mulliken	Hirshfeld	
T_BH-WSe2_	0.055e	0.030e	− 0.147e	− 0.2688e	2.125
T_BM-WSe2_	0.049e	0.1567e	− 0.147e	− 0.2690e	2.915
T_V-WSe2_	0.018e	0.0528e	− 0.017e	− 0.0292e	3.416

Table 3 presents intriguing findings, indicating a negative total charge on the CO_2_ molecule, suggesting an accumulation of electron density over the CO_2_ molecule. These electrons appear to be extracted from the Pt-atom, as evidenced by the negative value displayed in the CDD diagram. The impact of CO_2_ adsorption on the electronic structure (band structure, DOS, and PDOS) and electronic spin is presented in the supplementary information (Figs. S9, S11(d1–d3)). Furthermore, we performed calculations for the adsorption energies of the CO_2_ gas molecule on T_BH-WSe2_, T_BM-WSe2_, and T_V-WSe2_ configurations, resulting in values of − 0.5577 eV, − 0.5373 eV, and − 0.5777 eV, respectively. As it can be observed that there is slight deformation in the CO_2_ configuration after the optimization (for T_BH-WSe2_ and T_BM-WSe2_), due to the stronger adsorption energy and less adsorption distance.

### SO_2_ adsorption

**Figure 6:**
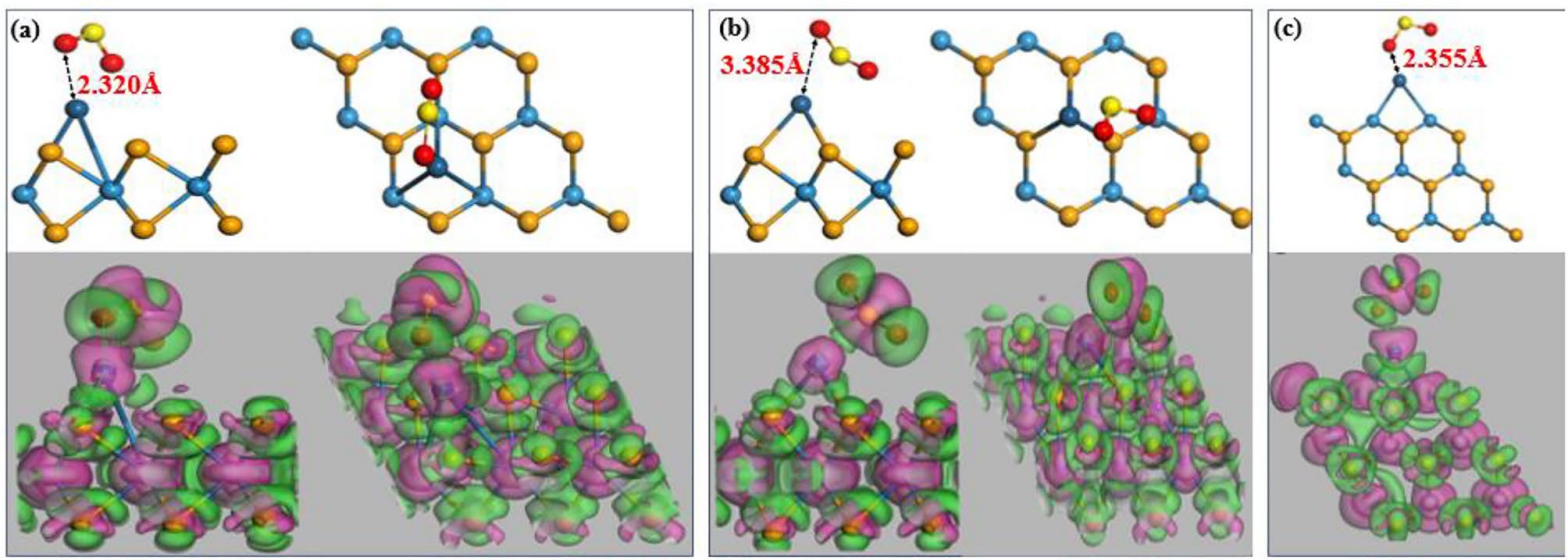
Optimal arrangement and associated charge density distribution after SO_2_ adsorption at three distinct locations where a Pt is functionalized: (**a**) the hollow site in the basal configuration, (**b**) positioned above the W atom in the basal configuration, and (**c**) situated along the vertical edge of WSe_2_. Electron depletion and accumulation are represented by the colour spectrum, with rosy signifying depletion and green signifying accumulation.

The adsorption configuration of the SO_2_ gas molecule is similar to the CO_2_ and NO_2_ gas molecules for the T_BH-WSe2_, T_BM-WSe2_, and T_V-WSe2_ as shown in Fig. 6a–c. In addition, there is a variation in S–O bond length (~ 0.036 Å) after adsorption as it can be seen in the CDD diagram. This variation is due to the stronger adsorption energy of these structures as aforementioned. Consequently, it implies that there is weak vdW interaction exhibited between Pt-atom and SO_2_ molecule. In T_BM-WSe2_, the Pt-atom interacts with the S atom, with an adsorption distance of 2.608 Å, exceeding the sum of relevant covalent radii (2.410 Å for Pt and S^48^). Conversely, in T_BH-WSe2_ and T_V-WSe2_, the Pt-atom interacts with the O atom, exhibiting adsorption distances of 2.355 Å and 2.320 Å, respectively, surpassing the sum of relevant covalent radii (2.020 Å for Pt and O^48^). The consistent observation across all three configurations reveals that the adsorption distances are greater than the sum of relevant covalent radii, indicating a physisorption mechanism, as corroborated by the CDD diagram (Fig. 6). Additionally, it is essential to emphasize that SO_2_ gas exhibits an oxidizing nature, resulting in the withdrawal of electrons from the system. To validate this phenomenon, we conducted an extensive analysis using Mulliken and Hirshfeld’s population analysis method, and the adsorption length data are presented in Table 4.

**Table 4:** Provides a tabulated overview of electron distribution and SO_2_ adsorption values for three distinct configurations.

System	Total charge over Pt		Total charge over SO_2_		Adsorption distance between Pt and O atom (in Å)
	Mulliken	Hirshfeld	Mulliken	Hirshfeld	
T_BH-WSe2_	0.125e	0.1417e	− 0.202e	− 0.1746e	2.355
T_BM-WSe2_	0.088e	0.0908e	− 0.093e	− 0.1899e	3.385
T_V-WSe2_	0.125e	0.1417e	− 0.202e	− 0.1746e	2.320

The findings from Table 4 revealed a negative total charge on the SO_2_ molecule, which implies the electron accumulation within the SO_2_ molecule. These electrons seem to be drawn from the Pt-atom, as evidenced by the negative value shown in the CDD diagram. Further details regarding the electronic structure analysis (including band structure, DOS, and PDOS) and electronic spin upon SO_2_ gas molecule adsorption can be found in the supplementary information (Figs. S10, S11(e1–e3)). Moreover, we obtained the adsorption energy of SO_2_ gas molecules to be − 0.6813 eV, − 0.7511 eV, and − 0.8391 eV for T_BH-WSe2_, T_BM-WSe2_, and T_V-WSe2_, respectively. The most promising results of T_V-WSe2_, which suggests a potent physisorption action, are particularly remarkable.

## Sensing mechanism

**Figure 7:**
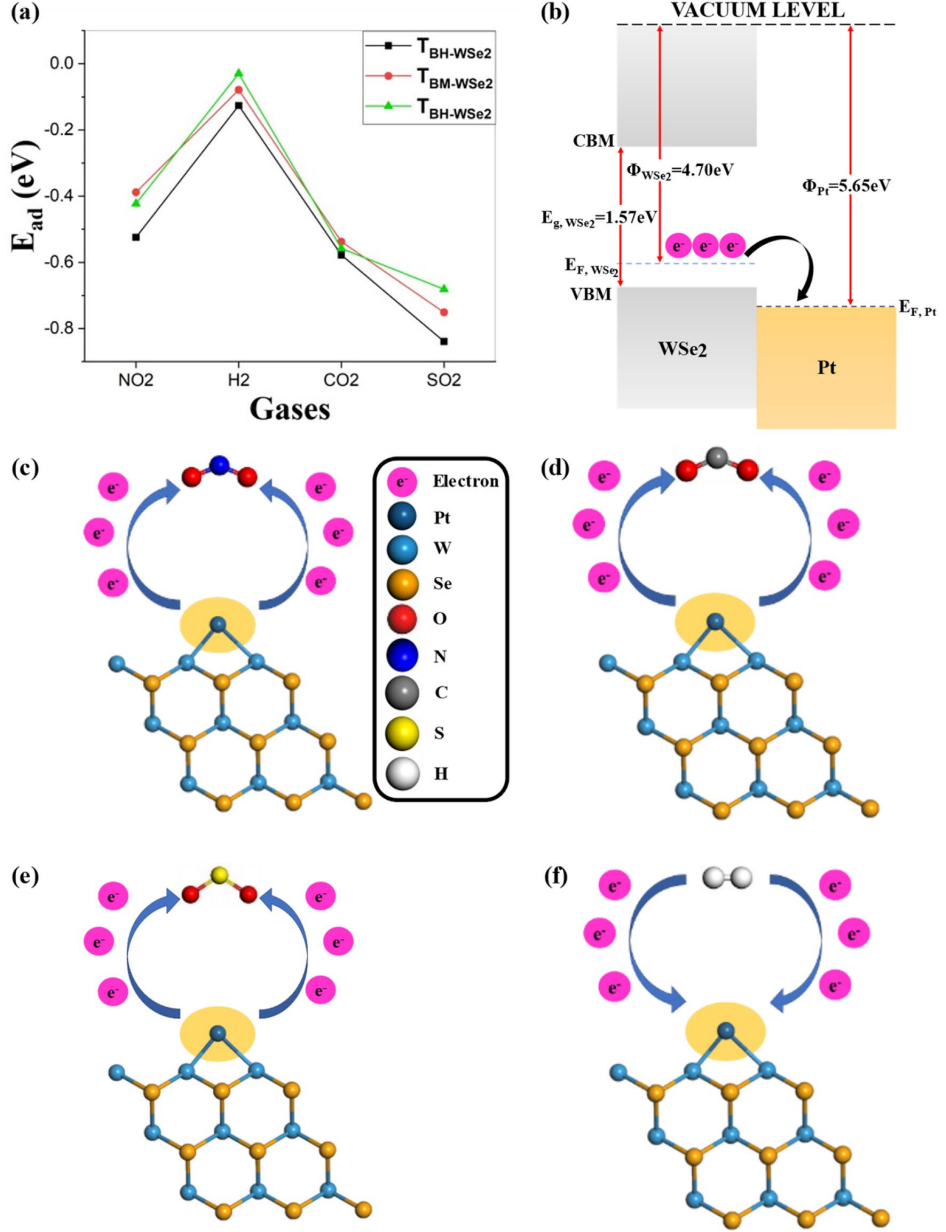
(**a**) Adsorption energy for three different configurations with respect to target gases, (**b**) the energy band diagram of the proposed structure. Schematic diagrams depicting the gas sensing mechanisms for (**c**) NO_2_, (**d**) CO_2_, (**e**) SO_2_, and (**f**) H_2_ gas molecules.

To interpret the sensing mechanism of Pt-WSe_2_ composites, we examined the molecular interaction and charge transfer at the interface. It can be observed that the adsorption energy (as mentioned in Table S1) is improved in T_V-WSe2_ configuration among all proposed structures indicating strong interaction between the target gas molecule and Pt. A negative adsorption energy value signifies that the adsorption process releases heat, making it exothermic. Moreover, the adsorption energy (Fig. 7a) value is also listed in Table S1. (Refer to Table S1 in the supporting information).

Moreover, to confirm the response of H_2_ due to higher binding capacity we have calculated the sensitivity. The electrical conductivity ($\sigma$) can be defined as^50^:

1 $$\sigma =ne\mu$$

Here, *e* and *µ* represent carrier charge and mobility (constant because of monolayer), respectively. And *n* denotes the carrier concentration and it is defined as^50^:

2 $$n \propto {e}^{\left(\frac{{-E}_{g}}{{2k}_{B}T}\right)}$$

where *E*_*g*_ represents the bandgap of the system. It can be seen that carrier concentration is dependent on the *E*_*g*_. The sensitivity (*S*) is defined as a relative change in resistance^51^:

3 $$S=\frac{\left|{R}_{Sys, Gas}-{R}_{Sys, Isolated}\right|}{{R}_{Sys, Gas}}$$

$$S= \frac{\left|\frac{1}{{\sigma }_{Sys,Gas}}-\frac{1}{{\sigma }_{Sys,Isolated}}\right|}{\frac{1}{{\sigma }_{Sys,Gas}}}$$

$$S=\frac{\left|{\sigma }_{Sys,Isolated}-{\sigma }_{Sys,Gas}\right|}{{\sigma }_{Sys,Gas}}$$

$$S=\left|\frac{{\sigma }_{Sys,Isolated}}{{\sigma }_{Sys,Gas}}-1\right|$$

Using Eq. (3), the sensitivity can also be represented as:

$$S= \left|\frac{{e}^{\left(\frac{{-E}_{{g}_{(Sys,Isolated)}}}{{2k}_{B}T}\right)}}{{e}^{\left(\frac{{-E}_{{g}_{(Sys,Gas)}}}{{2k}_{B}T}\right)}}-1\right|$$

4 $$S= \left|{e}^{\left(\frac{{E}_{{g}_{(Sys,Gas)}}{-E}_{{g}_{(Sys,Isolated)}}}{{2k}_{B}T}\right)}-1\right|$$

Equation (4) shows the simplified equation of *S* where, ${E}_{{g}_{(Sys,Gas)}}$, and ${E}_{{g}_{(Sys,Isolated)}}$ represents the energy band gap of the system with and without gas adsorption, respectively. According to Eq. (4), the sensitivity value for distinct target gas molecules (H_2_, NO_2_, CO_2_, and SO_2_) adsorbed T_BH-WSe2_, T_BM-WSe2_, and T_V-WSe2_ systems is tabulated in Table 5.

**Table 5:** Summarizes the sensitivity of the three configurations to the target gas molecule.

System	Gases	E_g(Sys,Gas)_ (eV)	Sensitivity (%)
T_BH-WSe2_	H_2_	0.7508	82.6
	NO_2_	0.7491	75.3
	CO_2_	0.7494	76.2
	SO_2_	0.7453	62.7
T_BM-WSe2_	H_2_	1.2682	84.2
	NO_2_	1.1801	66.5
	CO_2_	0.6466	75.5
	SO_2_	0.6645	65.5
T_V-WSe2_	H_2_	0.0954	92.1
	NO_2_	0.7512	82.4
	CO_2_	0.7518	84.5
	SO_2_	0.7468	67.6

Figure 7b–f represents the schematic of energy band structure and gas sensing mechanisms for T_V-WSe2_-based configuration towards NO_2_, CO_2_, SO_2_, and H_2_ gas molecule. As we can see from the energy band diagram when a Pt single atom functionalized over WSe_2_, there is a difference between the Fermi level of WSe_2_ and Pt i.e., work function (ϕ) of WSe_2_ is less than Pt due to which the electrons will transfer from WSe_2_ to Pt-atom until the Fermi levels of both the constituents (Pt and WSe_2_) lie at the equilibrium. During the adsorption of NO_2_, CO_2_ and SO_2_ molecules over Pt-WSe_2_ composite, gases will withdraw electrons due to their oxidizing nature. As it is also verified from the CDD, Mulliken, and Hirshfeld analysis as discussed in the aforementioned subsections. Upon NO_2_, CO_2_, and SO_2_ adsorption, there is a depletion of electrons over Pt due to which it possesses a positive value and accumulation of electrons over target gas molecule (NO_2_, CO_2_, and SO_2_) resulting in a negative value. Consequently, there is a depletion of charge carriers due to which the sensing material resistance gets modified. But, the H_2_ molecule has a reducing nature due to which it donates electrons to the sensing layer. This is also confirmed by the CDD, Mulliken, and Hirshfeld analysis. Upon H_2_ adsorption, there is the accumulation of electrons over Pt due to which it possesses a negative value, and depletion of electrons over H_2_ resulting in a positive value. As it can also be observed that there is elongation in the H–H bond after adsorption over Pt-WSe_2_ which confirms the spillover effect. As a result of this phenomenon, the H atom undergoes dissociation, leading to interactions with the WSe_2_ layer, ultimately resulting in an increase in sensitivity to H_2_ molecules. Furthermore, our computational results as mentioned in Table 5 affirm that the T_V-WSe2_ configuration exhibits higher sensitivity in the presence of H_2_ gas molecules. Moreover, we examine the ϕ(s) of pristine and Pt-decorated WSe_2_ monolayers under gas adsorption to comprehend their electron overflow behaviour. Figure S11 displays all of the systems’ computed ϕ values (see the Supplementary Information for details). Furthermore, to confirm sensing performance we employed the van’t-Hoff–Arrhenius expression shown in Eq. (5)^42^ and analyzed the recovery time (in s) which is presented in Table S1.

5 $$\tau ={A}^{-1}{e}^{\left(\frac{{-E}_{a}}{{k}_{B}T}\right)}$$

where A, k_B_, and T represent attempted frequency (~ 10^–12^ s^−1^)^42^, Boltzmann constant, and temperature, respectively. *E*_*a*_ denotes the potential barrier for desorption which is equivalent to adsorption energy. As it can be seen the recovery time for the T_V-WSe2_-based system to detect SO_2_ is maximum at low temperatures due to strong adsorption energy. Moreover, due to strong adsorption energy among all configurations, the adsorption and desorption are dependent on activation and deactivation energy, respectively. The deactivation energy depends on temperature hence the recovery time decreases with an increase in temperature. As it can be depicted in Table S1 all three proposed configurations have faster recovery time in the case of H_2_ gas molecules. In previous studies, it has been shown that the Pt loaded over 2D nanomaterial configuration is highly selective for the H_2_ gas molecule. And among these systems adsorption energy is comparatively stronger in the case of T_V-WSe2_-based H_2_ gas molecule as aforementioned in the result and discussion section. Upon H_2_ adsorption, there is an addition of energy states in the bandgap due to charge transport characteristics resulting in variation in the bandgap.

## Conclusions

In this work, we have decorated Pt over the basal and vertical edge of WSe_2_ and performed the DFT study for the gas sensing towards distinct target gases (H_2_, NO_2_, CO_2_, and SO_2_) using the Dmol^3^ package in Material Studio software. We have analyzed the electronic characteristics (bandstructure, DOS, CDD, and population analysis) of Pt decorated over the WSe_2_ system with and without adsorption of target gases. We have analyzed recovery times for distinct combinations in which the H_2_ adsorbed system shows quicker recovery in contrast to others. The rates of adsorption and desorption are directly influenced by the respective activation and deactivation energies, which exhibit a strong dependence on temperature. Moreover, the introduction of Pt onto WSe_2_ induces a spillover effect, substantiated by the elongation of the target gas molecule. In the T_V-WSe2_ configuration, there is an increased presence of W edge sites, serving as additional adsorption sites for the gas molecule. Significantly, these W edge sites exhibit higher binding capacity and larger surface area contributing to an enhanced sensitivity in T_V-WSe2_ towards the target gas molecules. T_V-WSe2_ shows an excellent sensitivity for the H_2_ molecule. The adsorption of gas molecules onto the sensing material causes a modulation in electrical conductivity, arising from the interaction between the adsorbed gas molecule and the sensing layer. Our work shows that the decoration of Pt over the vertically oriented WSe_2_ enhances the sensing performance significantly.

### Supplementary Information

Supplementary Information.

## Data Availability

The authors declare that the data supporting the findings of this study are available within the paper and its Supplementary Information files. Should any raw data files be needed in another format they are available from the corresponding author upon reasonable request.
