# Supplementary material for: A DFT study of superior adsorbate–surface bonding at Pt-WSe2 vertically aligned heterostructures upon NO2, SO2, CO2, and H2 interactions
Source: Sci Rep. 2024 Jul 8;14:15708. doi: 10.1038/s41598-024-65213-y (PMC11231162; doi:10.1038/s41598-024-65213-y)
Supplement: Supplementary file 1 — Supplementary Information. [file 41598_2024_65213_MOESM1_ESM.docx]

**Supporting Information for**

**A DFT Study of Superior Adsorbate–Surface Bonding at Pt-WSe_2_ Vertically Aligned Heterostructures upon NO_2_, SO_2_, CO_2_, and H_2_ Interactions**

Aditya Kushwaha, Neeraj Goel*

*Department of Electronics and Communication Engineering, Netaji Subhas University of Technology, Dwarka, New Delhi 110078, India*

Corresponding Author

^*^E-mail: [neeraj.goel@nsut.ac.in](mailto:neeraj.goel@nsut.ac.in)

Using equation 1 we have calculated the adsorption energy (*E_ad_)* [1, 2]:

$E_{ad}=E_{{ad}_{System/Gas}}-E_{{ad}_{System}}-E_{{ad}_{Gas}}$ (1)

Here, the adsorption energy of the system with and without gas is denoted by $E_{{ad}_{System/Gas}}$ and $E_{{ad}_{System}}$, respectively. While the $E_{{ad}_{Gas}}$, represents the adsorption energy of the target gases. Utilizing the equation 2, the "CDD" for the adsorption configuration (*Δρ*) is ascertained [3]:

$\Delta\rho={\Delta\rho}_{System/Gas}-{\Delta\rho}_{System}-{\Delta\rho}_{Gas}$ (2)

Where, ${\Delta\rho}_{System/Gas}$ denotes charge densities of the system in the presence of gas while ${\Delta\rho}_{System}$ and ${\Delta\rho}_{Gas}$ represents charge densities of isolated system and gas, respectively.

Fig. S1a and S1b exhibit the optimized configuration of T_BH-WSe2_, revealing structure's bandgap and DOS are measured at 0.720 eV (Fig. S1c and S1d). Similarly, Fig. S2a and S2b showcase the optimized configuration of T_BM_-_WSe2_. According to the bandgap and DOS diagram for T_BM-WSe2_ are observed to be 1.237 eV (Fig. S2c and S2d). Fig. S3a and S3b display the stable configuration of T_V-WSe2_ after optimization. Notably, this configuration exhibits a drastically lower bandgap of 0.062 eV compared to the other configurations, as depicted in Fig. S3c and S3d. When Pt-NP is functionalized onto the WSe_2_ monolayer, their electronic orbitals hybridize with those of the surrounding W and Se atoms. This creates new electronic states within the bandgap of pristine WSe_2_, which results in lowering the bandgaps. There are slight bond length variations post-optimization. The bandgap value has been decreased due to the introduction of Pt-NP. This finding underscores the dramatic influence of Pt-NP placement on the electronic properties of WSe_2_, highlighting the potential for tailoring the system's functionality by manipulating the decoration site. The negative value adsorption energy indicates that it is an exothermic process. After optimization, the bond length between Pt-NP and W-atom for T_BH-WSe2_, and T_V-WSe2_ are 3.917 Å and 2.802 Å. While in the case of T_BM-WSe2_ the bond length between Pt and Se atom is 2.429 Å.


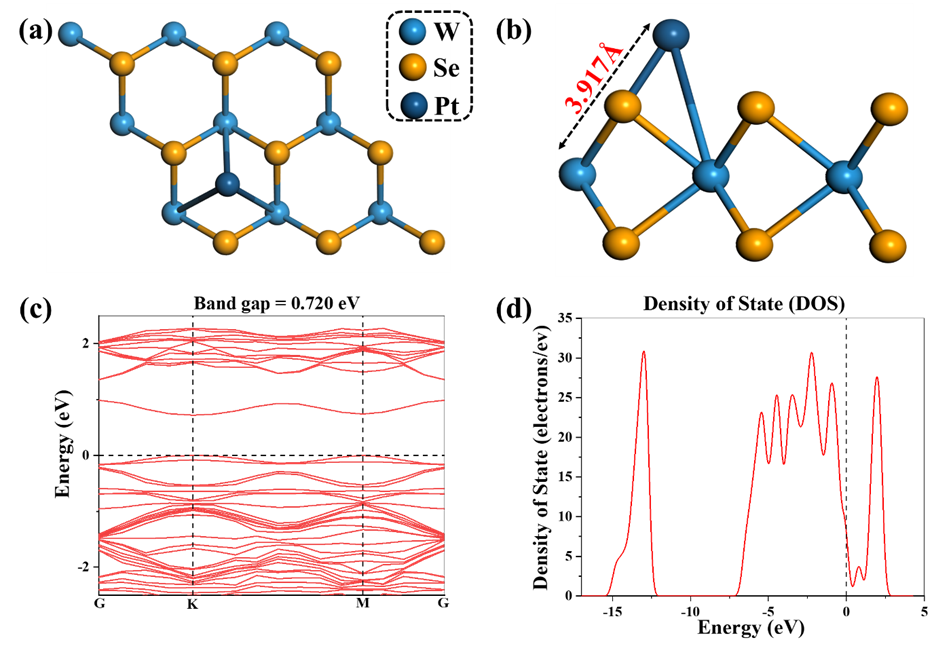


**Fig. S1 a)** Top view, and **b)** side view of the platinum atom positioned above the hollow hexagonal site in the 3x3 WSe_2_ basal plane configuration (T_BH-WSe2_). **c)** The electronic band structure of the T_BH-WSe2_ system, and **d)** density of states (DOS). The horizontal and vertical dashed lines mark the Fermi level in the band structure and DOS plots, respectively.


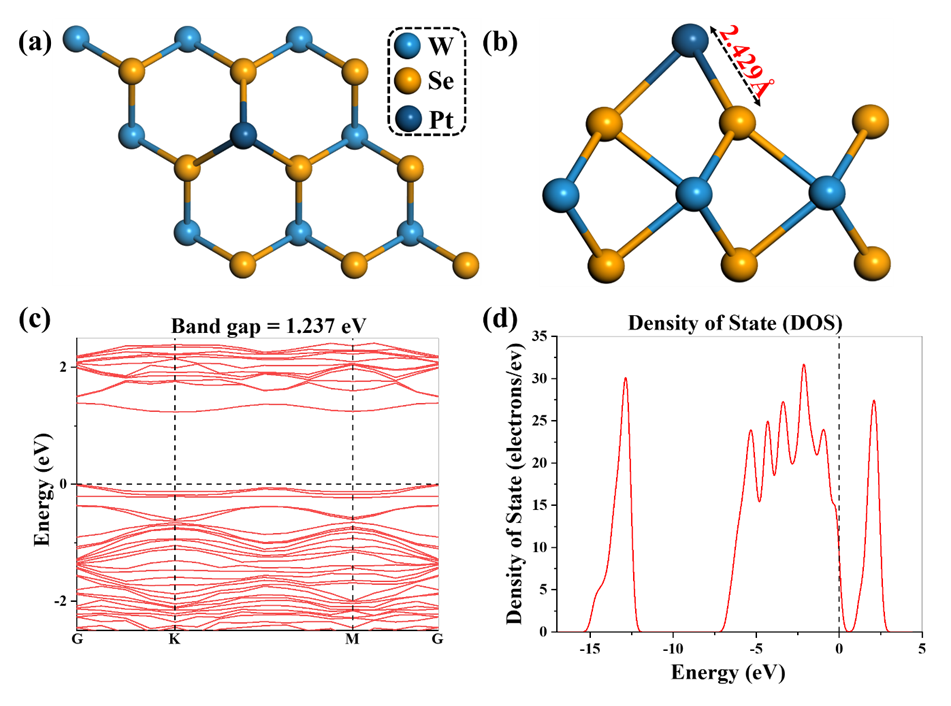


**Fig. S2 a)** Top view, and **b)** side view of the platinum atom decorated over the W atom in WSe_2_ basal plane configuration (T_BM-WSe2_). **c)** The electronic band structure of the T_BM-WSe2_ system, and **d)** density of states, with Fermi levels indicated by horizontal and vertical dashed lines in the respective plots.


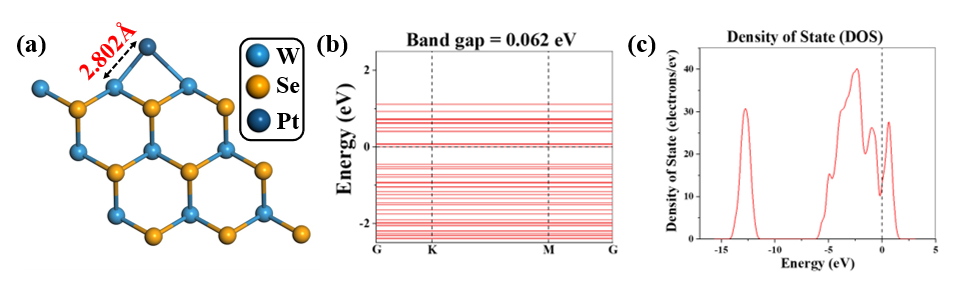


**Fig. S3** **a)** Front view of the platinum atom decorated at the vertical edge of WSe_2_ (T_V-WSe2_). **b)** The electronic band structure, and **c)** density of states for the T_V-WSe2_ system are presented, with Fermi levels marked by horizontal and vertical dashed lines in the corresponding plots.

Furthermore, for the charge distribution investigation, we have done Mulliken and Hirshfeld analysis. Fig. S4 depicts the CDD for pristine WSe_2_, T_BH-WSe2_, T_BM-WSe2_, and T_V-WSe2_. In the case of T_BH-WSe2_, T_BM-WSe2_, and T_V-WSe2_ the Mulliken and Hirshfeld analysis depicts the depletion of electrons at Pt-NP i.e., Pt-NP possesses positive charge. According to the Mulliken and Hirshfeld calculation of T_BH-WSe2_, T_BM-WSe2_, and T_V-WSe2_ the charge over Pt-NP is 0.050 e & 0.030 e, 0.056 e & 0.031 e, and 0.062 e & 0.037 e, respectively as shown in Fig. S4b-S4d. In previous studies, it has been shown that Pt-NP promotes catalytic oxidation, and due extraction of electrons from nanosheets which causes a hole accumulation layer [4]. In Fig. Fig. S4, rosy and green colours represent the electron depletion and accumulation, respectively.


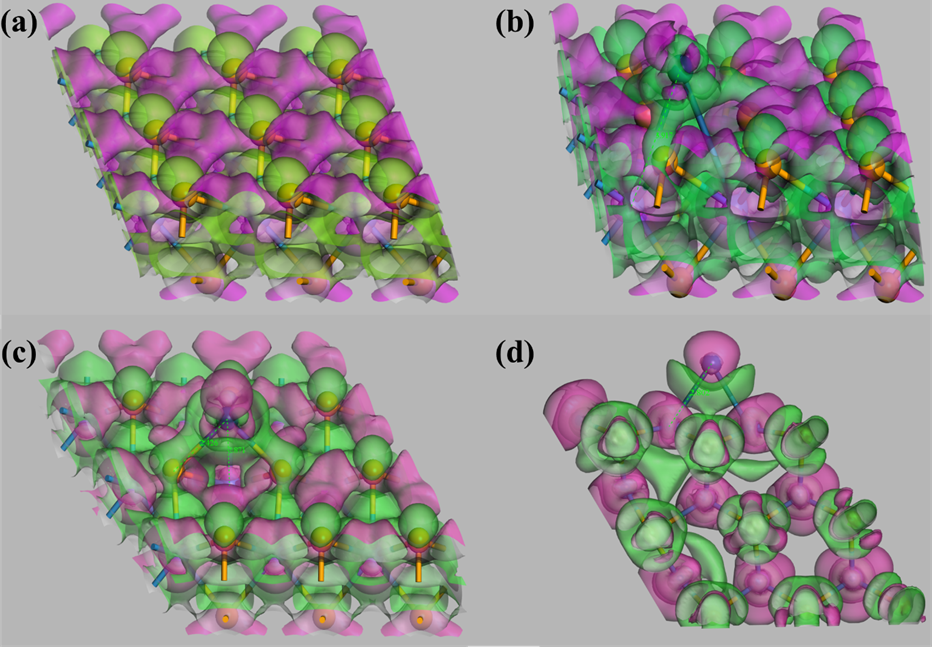


**Fig. S4** Charge density difference of **a)** the monolayer of 3x3 WSe_2_, **b)** a platinum atom functionalized above the hollow hexagonal site within the basal plane of 3x3 WSe_2_ (T_BH-WSe2_), **c)** the Pt-NP decorated over the W atom in the basal plane of WSe_2_ (T_BM-WSe2_), and **d)** the Pt-NP positioned along the vertical edge of WSe_2_ (T_V-WSe2_). We represent electron depletion and accumulation using rosy and green colours, respectively.

The thermal stability of T_BH-WSe2_, T_BM-WSe2_, and T_V-WSe2_ configurations was evaluated through molecular dynamics (MD) simulations performed at 500 K for 1 ps with a timestep of 1 fs. Fig. S5a-S5c illustrates the results. The morphology of T_BH-WSe2_ and T_V-WSe2_ monolayers exhibits minimal deformation within a temperature range of ~300 K, suggesting good structural stability. In contrast, T_BM-WSe2_ maintains its structure up to ~900 K, indicating superior thermal stability at high temperatures. Additionally, minimal energy fluctuations were observed across the entire temperature range for all Pt-decorated WSe_2_ monolayers (T_BH-WSe2_, T_BM-WSe2_, and T_V-WSe2_). This suggests excellent thermal stability for the Pt decoration on the WSe_2_ surface (Fig. S5a-S5c). Furthermore, due to its superior stability and enhanced adsorption energy compared to other configurations, the T_V-WSe2_ monolayer was chosen for further analysis of its dynamical properties. Phonon dispersion calculations were performed to investigate the vibrational modes of T_V-WSe2_. The results are presented in Fig. S5d. The analysis reveals several key vibrational modes at the G-point (gamma point): A' (~260 cm^-1^) and E'' (~175 cm^-1^) are classified as Raman-active, while A_2_'' (300 cm^-1^) is infrared-active, and E' (~260 cm^-1^) exhibits dual Raman and infrared activity, which shows good agreement with previously reported work [5].


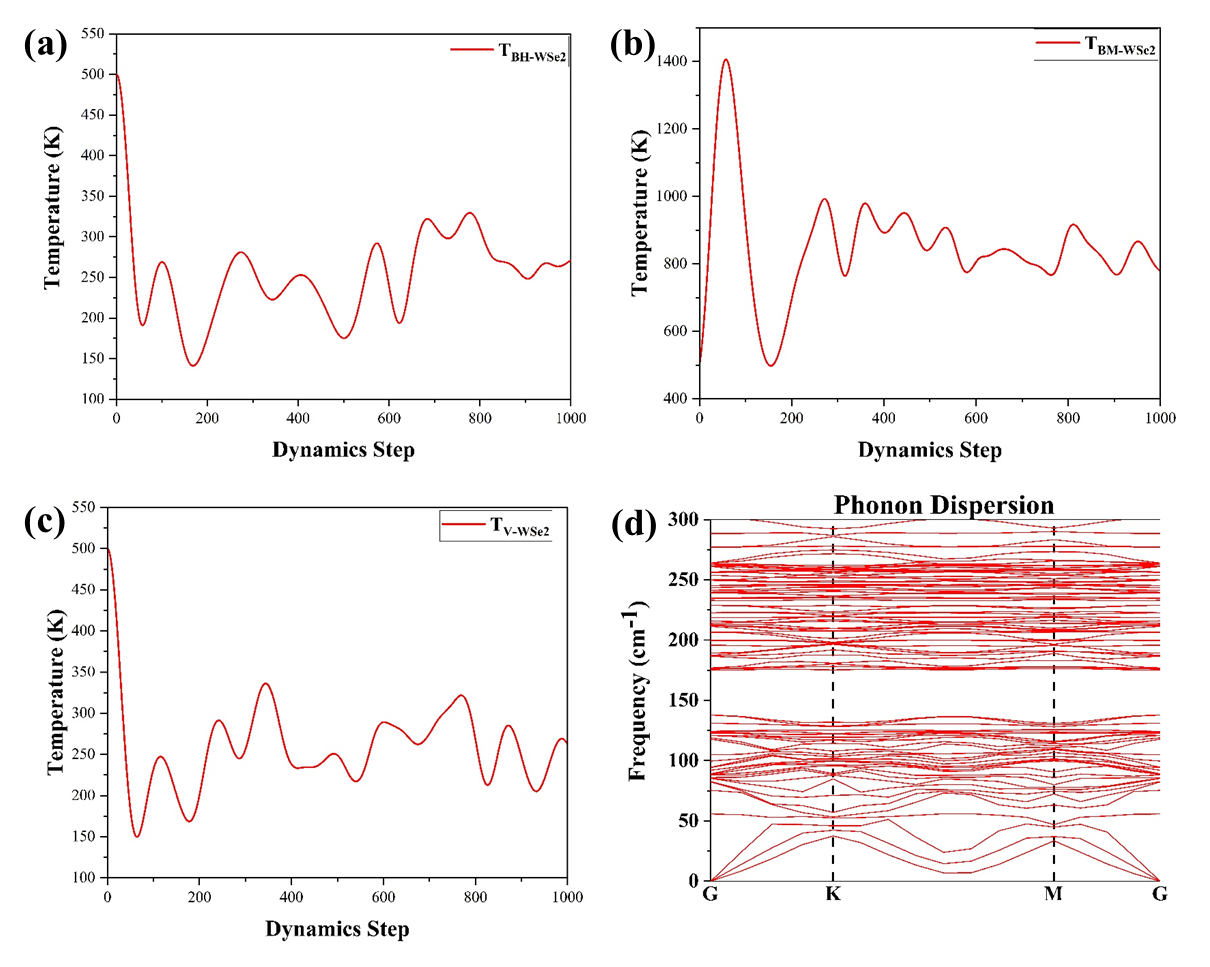


**Fig. S5** Molecular dynamics curve for **(a)** T_BH-WSe2_, **(b)** T_BM-WSe2_, and **(c)** T_V-WSe2_. **(d)** Phonon dispersion graph of T_V-WSe2_.

The initial gas-phase geometries for H₂, CO₂, NO₂, and SO₂ were obtained based on the crystallographic point groups of their respective stable conformers: I4/mmm (H₂), Im3̅ (CO₂), P4₂/mnm (NO₂), and Aea2 (SO₂). Subsequent density functional theory (DFT) calculations were employed to optimize the molecular geometries. In this optimization process, the atomic positions were iteratively adjusted to minimize the total energy of the molecule, effectively leading to the most stable conformation in the gas phase. Fig. S6 displays the optimized configurations of H_2_, NO_2_, CO_2_, and SO_2_ gas molecules. In Fig. S6a and S6b, the linear structures of H_2_ and CO_2_ molecules are illustrated, with bond lengths of 0.760 Å for H-H and 1.193 Å for C-O. The O-C-O angle in Fig. S5b is 179.457°. Fig. S6c and S6d show the optimized structures of NO_2_ and SO_2_, with bond lengths of 1.258 Å for C-O and 1.569 Å for S-O. The angles between O-N-O and O-S-O atoms are 122.241° and 112.987°, respectively. The observed bond lengths align with previous studies [6, 7]. Notably, the H_2_ and CO_2_ molecules exhibit shorter and linear structures due to sp hybridization, resulting in stronger electronegativity. In contrast, NO_2_ and SO_2_ molecules with sp^2^ hybridization show comparatively less electronegativity.


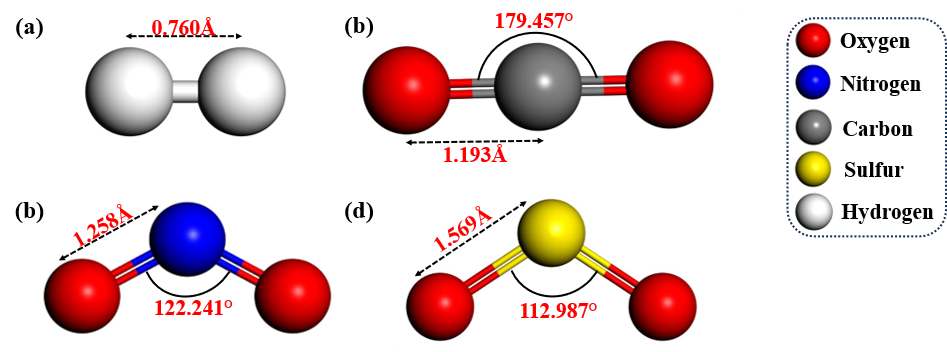


**Fig. S6** Most stable and optimized structure of gases **a)** H_2_, **b)** CO_2_, **c)** NO_2_, **d)** SO_2_.

The bandgaps, density of states (DOS), and projected density of states (PDOS) of the Pt-WSe_2_ systems exhibit varied enhancements upon H_2_ gas adsorption, for the T_BH-WSe2_, T_BM-WSe2_, and T_V-WSe2_ configurations, respectively (refer to Fig. S7). The increase in bandgap after H_2_ interaction suggests a decrease in the electrical conductivity of all three systems. This observation is supported by the analysis of DOS and PDOS deformations. Specifically, the DOS plot is expected to show an increase near the Fermi level, indicating the introduction of additional electronic states due to the donated electrons. Additionally, the PDOS for the H atom is anticipated to exhibit a distinct peak near the -5 eV energy level. Furthermore, the robust hybridization of orbitals around the Fermi level suggests significant orbital interaction between the gas molecule and the sensing material, indicating strong interaction between the two entities.

The bandgaps, DOS, and PDOS of the Pt-WSe_2_ systems demonstrate variation upon interaction with NO_2_, CO_2_, and SO_2_ gases for the T_BH-WSe2_, T_BM-WSe2_, and T_V-WSe2_ configurations, respectively (see Fig. S8-S10). This change in bandgap following interaction with the target gas molecules (NO_2_, CO_2_, and SO_2_) implies alterations in the electrical conductivity of all three systems. This observation is substantiated by analyzing the deformations in the DOS and PDOS. Specifically, the DOS plot is expected to exhibit variations near the Fermi level, indicating the introduction of additional electronic states due to electron withdrawal. Moreover, the PDOS for the target gas molecule demonstrates strong hybridization with the Pt orbitals, suggesting a robust interaction between the gas molecule and the Pt-WSe_2_ system.

When a gas molecule interacts with the surface of a material, there can be a transfer of electrons between them. This can happen through chemisorption or even strong physisorption. The redistribution of charge and hybridization of orbitals can introduce new energy levels within the band gap, effectively vary the energy difference between the valence and conduction bands [8, 9].


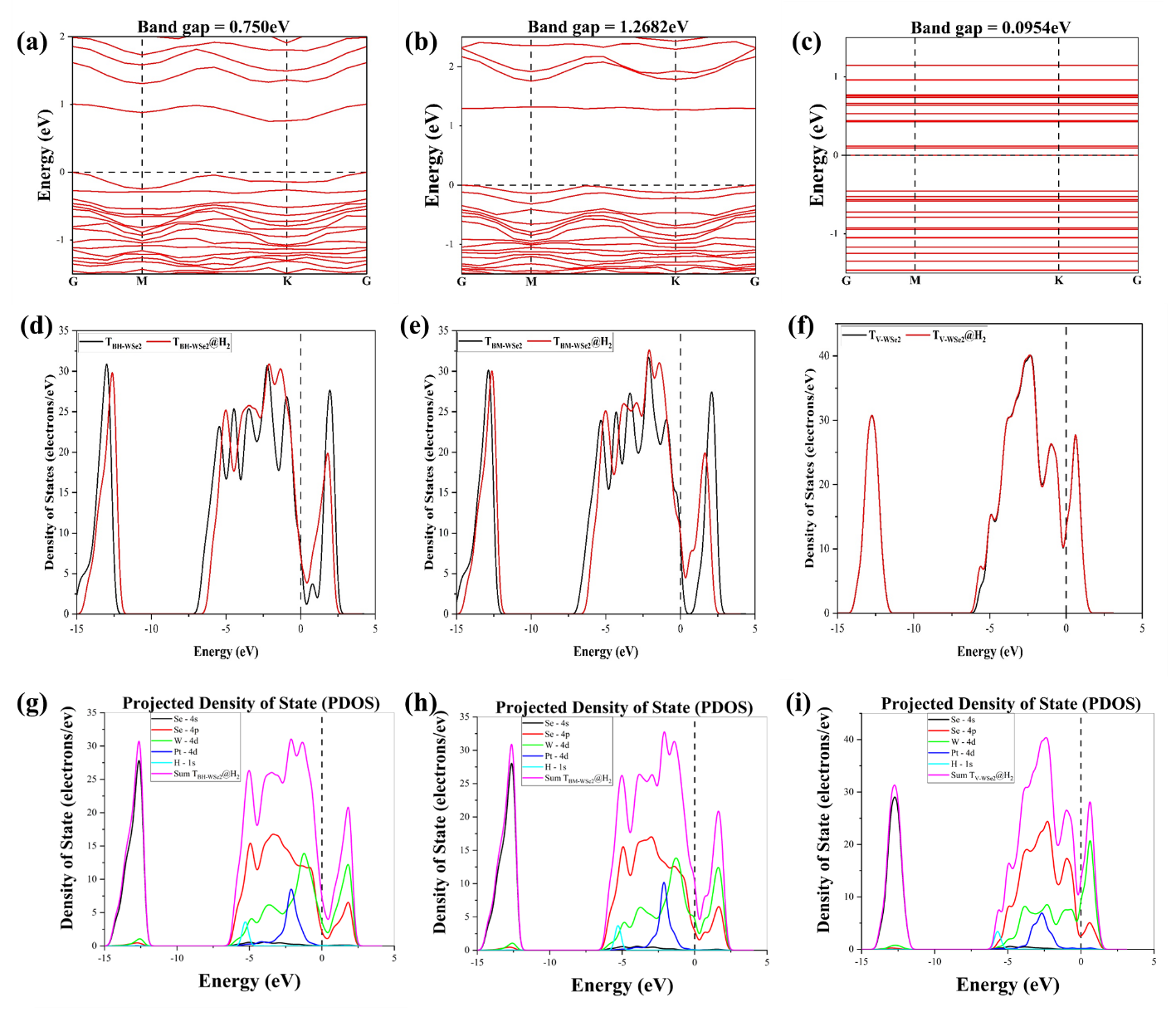


**Fig. S7** Investigation of **(a-c)** band gap, **(d-f)** density of states (DOS), and **(g-i)** projected density of states (PDOS) for H_2_ adsorption in the T_BH-WSe2_, T_BM-WSe2_, and T_V-WSe2_ configurations.


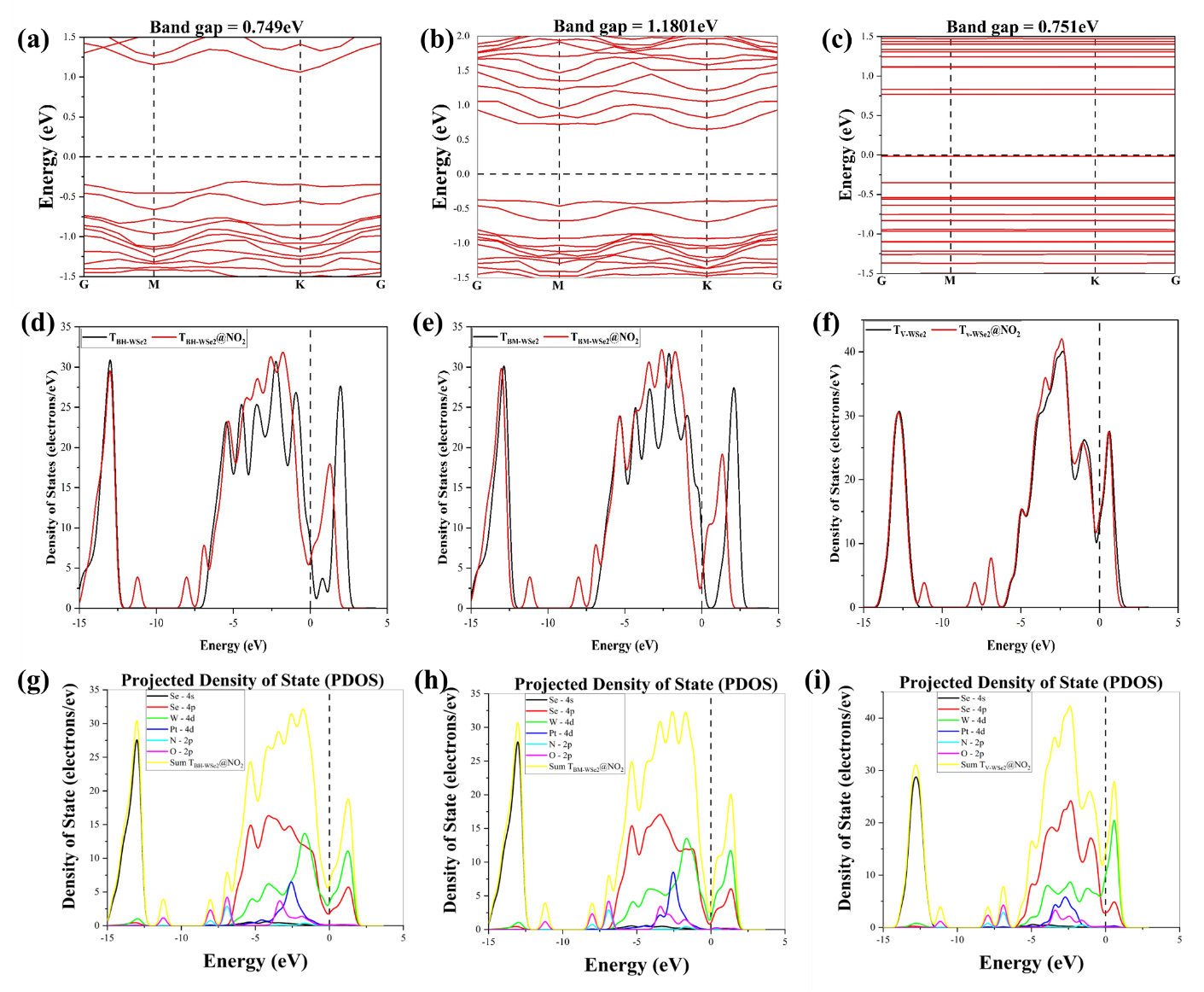


**Fig. S8** Exploring the **(a-c)** band gap, **(d-f)** density of states (DOS), and **(g-i)** projected density of states (PDOS) for NO_2_ adsorption in the T_BH-WSe2_, T_BM-WSe2_, and T_V-WSe2_ configurations.


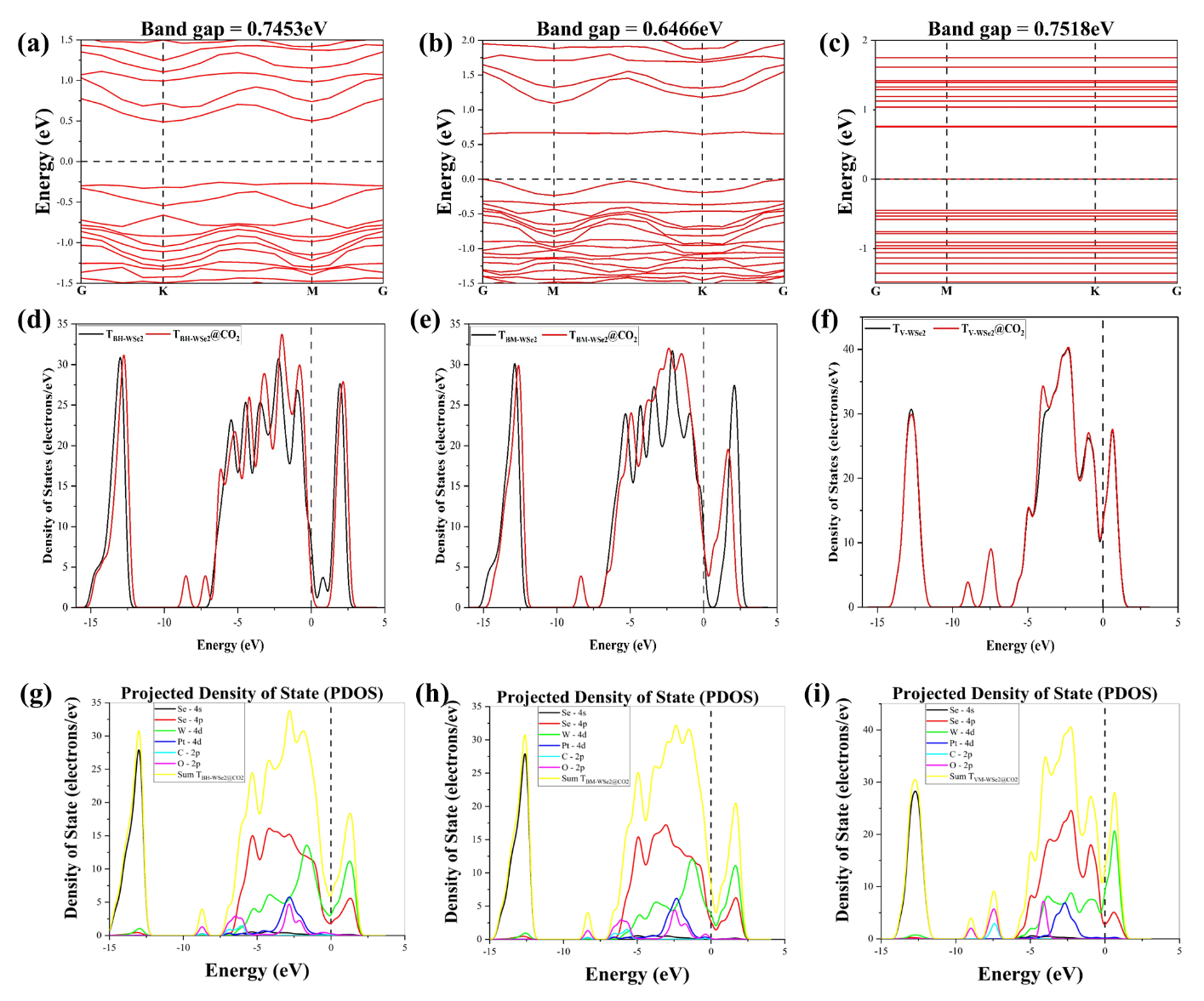


**Fig. S9 (a-c)** Band gap, **(d-f)** density of states (DOS), and **(g-i)** projected density of states (PDOS) analysis of CO_2_ adsorption on T_BH-WSe2_, T_BM-WSe2_, and T_V-WSe2_ systems.


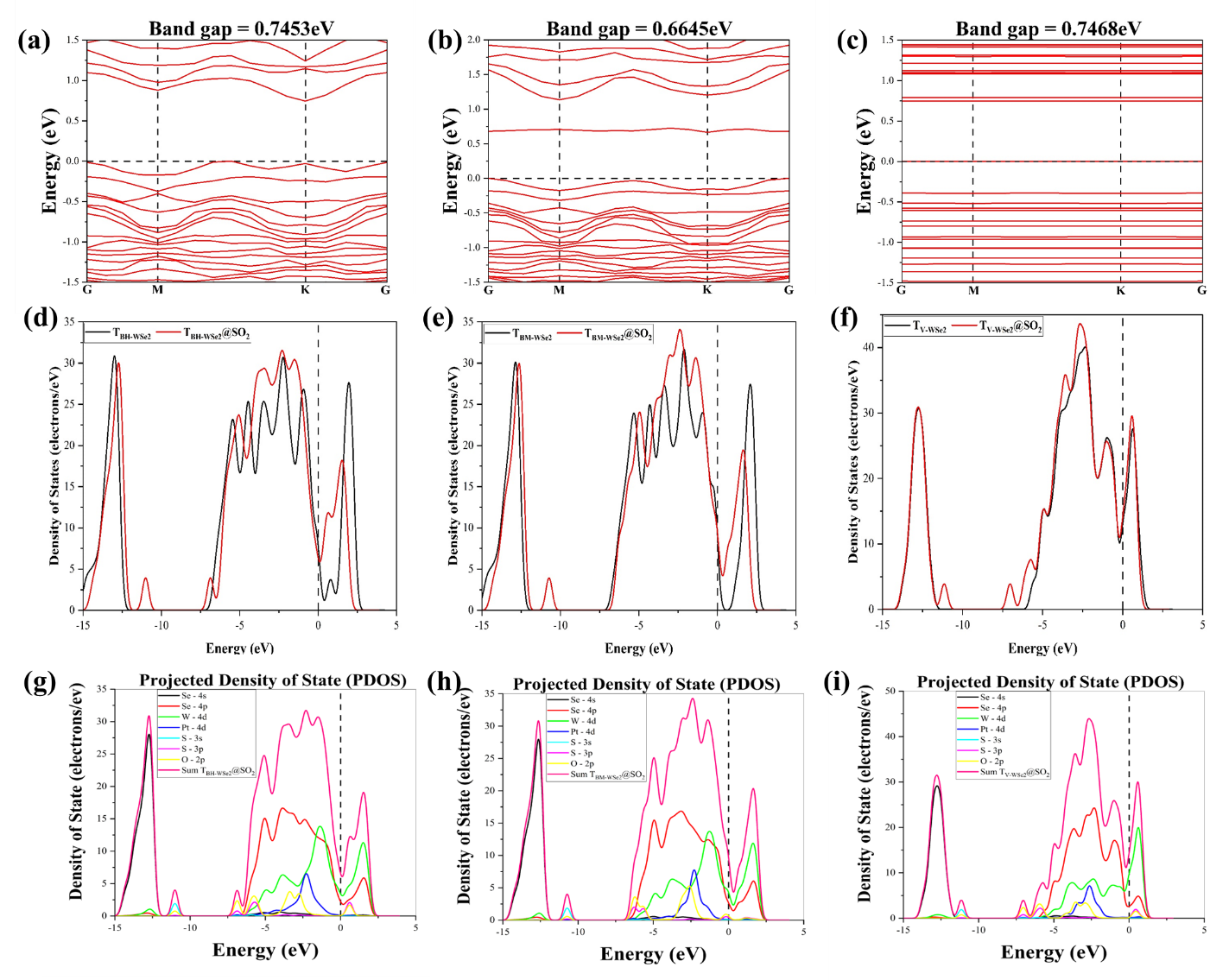


**Fig. S10** The analysis of **(a-c)** band gap, **(d-f)** density of states (DOS), and **(g-i)** projected density of states (PDOS) for SO_2_ adsorption on T_BH-WSe2_, T_BM-WSe2_, and T_V-WSe2_ systems.

**Investigating Electron Spin in Pt-Decorated WSe_2_ upon Gas Adsorption**

Our study employed total density of states (TDOS) analysis to investigate the influence of gas molecule adsorption (H_2_, NO_2_, CO_2_, and SO_2_) on the electronic spin properties of Pt-decorated WSe_2_ (Pt-WSe_2_). Notably, pristine Pt-WSe_2_ configurations exhibited symmetrical spin-up and spin-down channels in their TDOS profiles (Fig. S11(a1-a3)), signifying a non-magnetic character. This characteristic was preserved for Pt-WSe_2_ after H_2_ adsorption, as evidenced by the continued symmetry observed in the spin channels (Fig. S11(b1-b3)). In contrast, a significant shift was observed upon NO_2_ adsorption. The TDOS revealed an asymmetry in the spin-up and spin-down channels at specific energies (~11 eV) for all three Pt-WSe_2_ configurations (Fig. S11(c1-c3)). This indicates that NO_2_ adsorption induces partial spin polarization of the atomic orbitals, suggesting a transition to a magnetic configuration for Pt-WSe_2_ with adsorbed NO_2_ molecules. Interestingly, Pt-WSe_2_ retained its non-magnetic character following CO_2_ and SO_2_ adsorption. The symmetrical spin-up and spin-down channels observed in the TDOS profiles for both cases (Fig. S11(d1-d3) and Fig. S11(e1-e3)) support this conclusion.

**
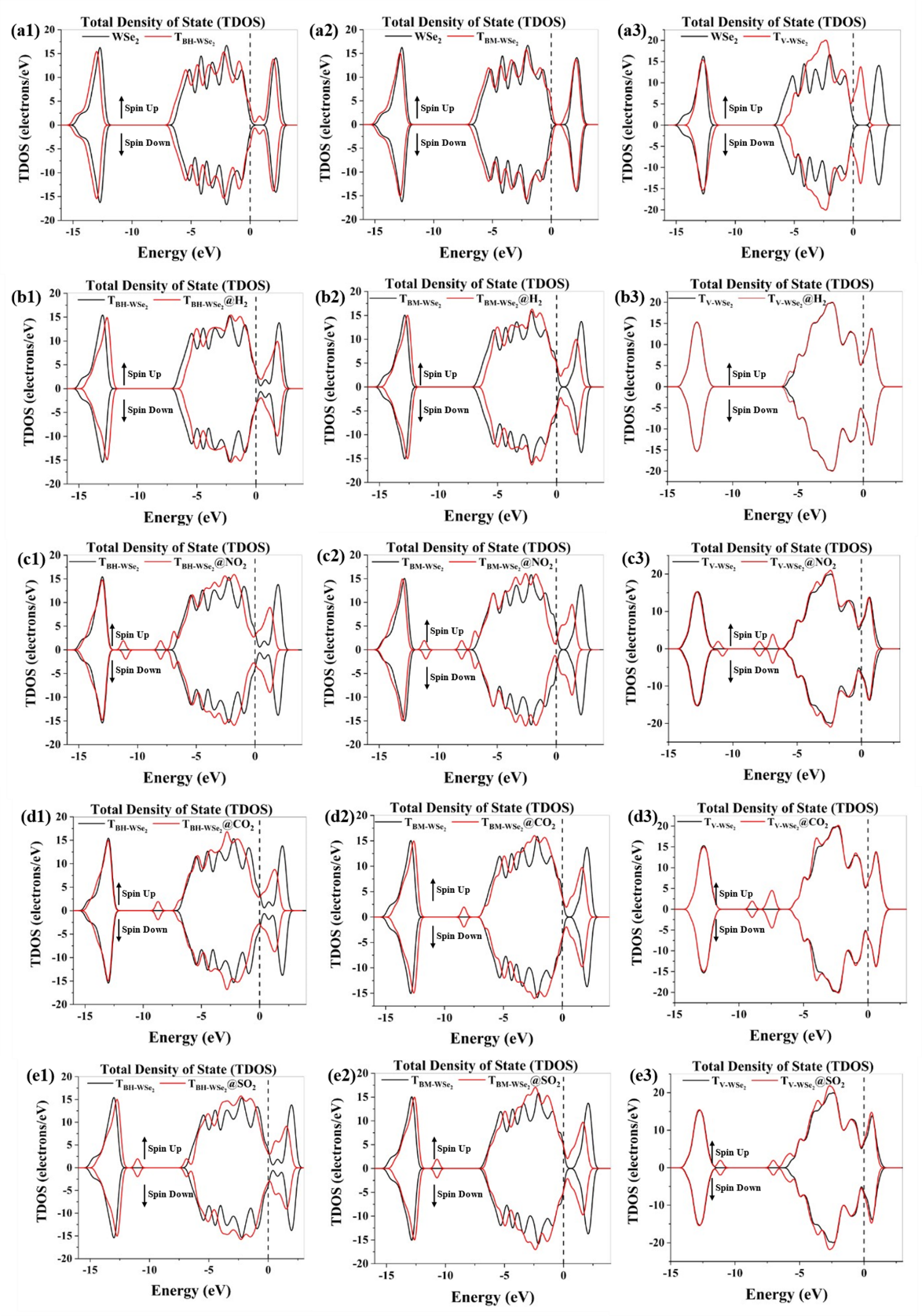
**

**Fig. S11** Total density of states (TDOS) for Pt-decorated WSe_2_ configurations. Panels a1-a3 depict the TDOS for three Pt positions: **a1)** over the hallow hexagonal site (T_BH-WSe2_), **a2)** atop a W atom (T_BM-WSe2_), and **a3)** at the vertical edge (T_V-WSe2_) of the WSe_2_ monolayer. The influence of gas molecule adsorption on the TDOS for each Pt-WSe_2_ configuration: **b1-b3)** H_2_, **c1-c3)** NO_2_, **d1-d3)** CO_2_, and **e1-e3)** SO_2_ molecules. The dashed line shows the Fermi level.

Determined by orbital localization, the work function (Φ) plays a crucial role in establishing the charge transfer, and molecular adsorption capacity, as depicted in Fig. S12. The T_BH-WSe2_, T_BM-WSe2_, and T_V-WSe2_ structures exhibit Φ values of 4.92526 eV, 5.03410 eV, and 4.87084 eV, respectively, while the pure WSe_2_ monolayer is predicted to have a Φ of 5.14295 eV. Notably, the introduction of Pt decoration appears to enhance electron realization at the vacuum level for the WSe_2_ monolayer, as evidenced by a slight reduction in the Φ upon Pt insertion into the monolayer. Substantial differences in Φ are observed upon gas adsorption across various configurations. For instance, in the T_BH-WSe2_ configuration, the Φ changes to 4.81641 eV, 5.03410 eV, 5.41506 eV, and 4.81641 eV following H_2_, NO_2_, CO_2_, and SO_2_ adsorption, respectively. This corresponds to changes of 2.209%, 2.209%, 9.944%, and 2.209% for H_2_, NO_2_, CO_2_, and SO_2_ adsorption, respectively. Similarly, for the T_BM-WSe2_ configuration, the Φ changes to 4.97968 eV, 5.38785 eV, 5.41506 eV, and 5.33343 eV following H_2_, NO_2_, CO_2_, and SO_2_ adsorption, respectively, corresponding to changes of 1.081%, 7.027%, 7.567%, and 5.945% for H_2_, NO_2_, CO_2_, and SO_2_ adsorption, respectively. Furthermore, for the T_V-WSe2_ configuration, the Φ changes to 5.14295 eV, 5.41506 eV, 5.44228 eV, and 5.36064 eV following H_2_, NO_2_, CO_2_, and SO_2_ adsorption, respectively, corresponding to changes of 5.586%, 11.173%, 11.731%, and 10.055% for H_2_, NO_2_, CO_2_, and SO_2_ adsorption, respectively. The significant Φ variations upon exposure to various gases demonstrate the potential of Pt-decorated WSe_2_ monolayers as Φ-type gas sensors, particularly for the detection of target gas molecule [10].


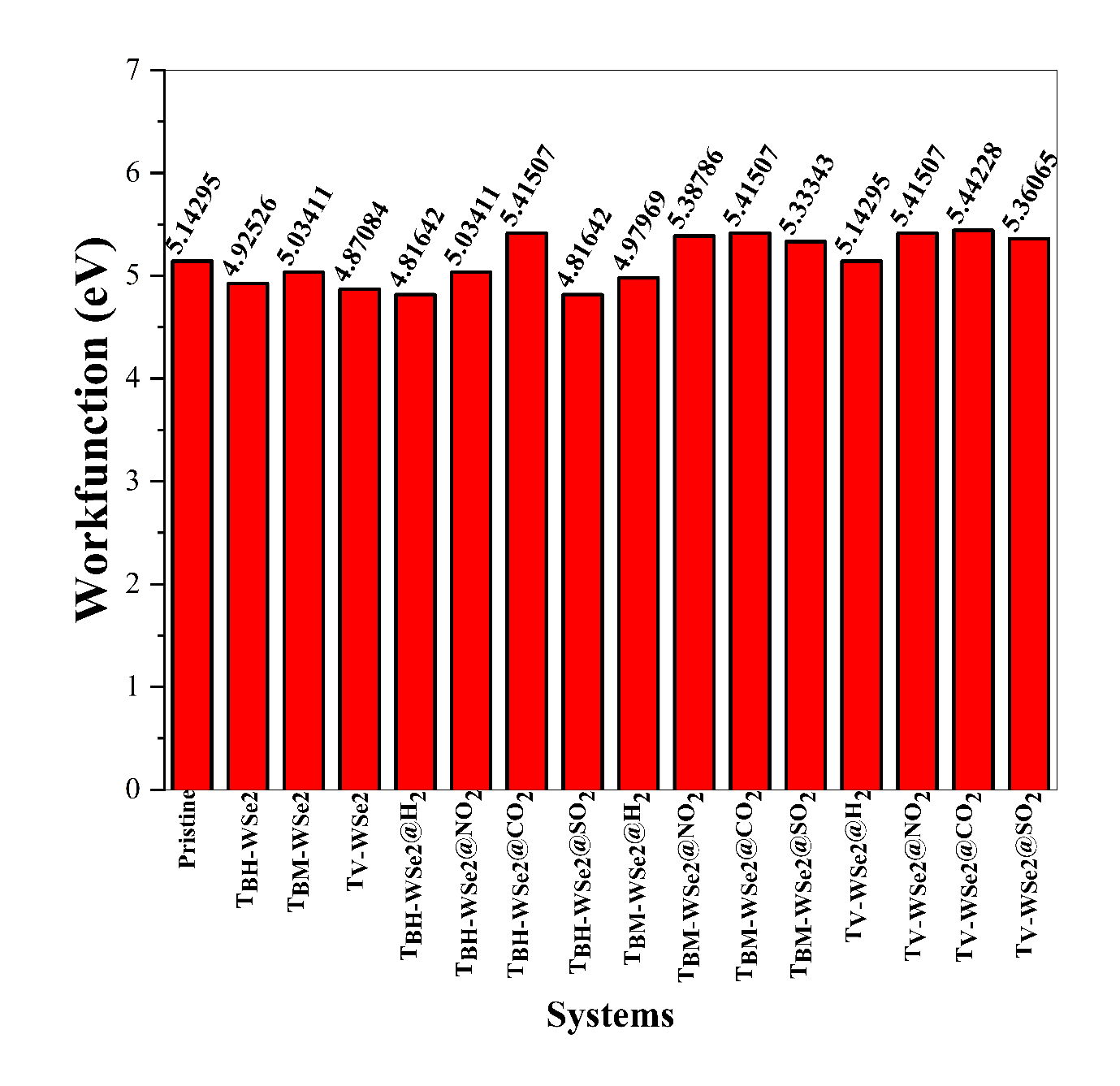


**Fig. S12** The work function of gas-adsorbed Pt-decorated and pristine WSe_2_ monolayers is presented in this graph.

**Table S1.** Presents a comprehensive comparison of adsorption energy (*E_ad_)* and recovery time(*τ)* at various temperatures for three proposed systems designed to target specific gas molecules.

| System | Gases | E_ad_ (eV) | Recovery Time (*τ* in sec) | | |
| --- | --- | --- | --- | --- | --- |
|  |  |  | **298K** | **348K** | **398K** |
| T_BH-WSe2_ | H_2_ | -0.0293 | 3.13E-12 | 2.66E-12 | 2.35E-12 |
|  | NO_2_ | -0.4227 | 1.41E-05 | 1.32E-06 | 2.25E-07 |
|  | CO_2_ | -0.5577 | 0.0027121 | 1.20E-04 | 1.16E-05 |
|  | SO_2_ | -0.6813 | 0.33338 | 0.0073679 | 4.24E-04 |
| T_BM-WSe2_ | H_2_ | -0.0788 | 2.16E-11 | 1.39E-11 | 9.97E-12 |
|  | NO_2_ | -0.3879 | 3.65E-06 | 4.16E-07 | 8.19E-08 |
|  | CO_2_ | -0.5373 | 0.0012256 | 6.06E-05 | 6.38E-06 |
|  | SO_2_ | -0.7511 | 5.0480259 | 0.0755023 | 0.0032463 |
| T_V-WSe2_ | H_2_ | -0.1261 | 1.36E-10 | 6.71E-11 | 3.96E-11 |
|  | NO_2_ | -0.5243 | 7.38E-04 | 3.93E-05 | 4.36E-06 |
|  | CO_2_ | -0.5777 | 0.0059095 | 2.33E-04 | 2.07E-05 |
|  | SO_2_ | -0.8391 | 155.23624 | 1.4192449 | 0.0422102 |

**References**

[1] J. Wang, X. Zhang, L. Liu, Z. Wang, Dissolved gas analysis in transformer oil using Ni-Doped GaN monolayer: A DFT study, Superlattices and Microstructures, 159(2021) 107055.

[2] H. Sun, L.-Q. Tao, T. Li, X. Gao, T. Sang, Y. Li, et al., TiO2–Doped GeSe Monolayer: A highly selective gas sensor for SF6 decomposed species detection based on DFT method, Applied Surface Science, 572(2022) 151212.

[3] M. Jiang, K. Xu, N. Liao, H. Zhou, DFT investigation on highly selective NO2 sensing properties of MnPS3, Applied Surface Science, 543(2021) 148846.

[4] R. Wadhwa, A. Kumar, R. Sarkar, P.P. Mohanty, D. Kumar, S. Deswal, et al., Pt Nanoparticles on Vertically Aligned Large-Area MoS2 Flakes for Selective H2 Sensing at Room Temperature, ACS Applied Nano Materials, 6(2023) 2527-37.

[5] K. Yuan, X. Zhang, L. Li, D. Tang, Effects of tensile strain and finite size on thermal conductivity in monolayer WSe 2, Physical Chemistry Chemical Physics, 21(2019) 468-77.

[6] M.D. Mohammadi, H. Louis, U.G. Chukwu, S. Bhowmick, M.E. Rasaki, G. Biskos, Gas-Phase Interaction of CO, CO2, H2S, NH3, NO, NO2, and SO2 with Zn12O12 and Zn24 Atomic Clusters, ACS omega, 8(2023) 20621.

[7] H. Van Ngoc, K.D. Pham, First-principles study on N2, H2, O2, NO, NO2, CO, CO2, and SO2 gas adsorption properties of the Sc2CF2 monolayer, Physica E: Low-dimensional Systems and Nanostructures, 141(2022) 115162.

[8] J. Di, J. Xiong, H. Li, Z. Liu, Ultrathin 2D photocatalysts: electronic‐structure tailoring, hybridization, and applications, Advanced materials, 30(2018) 1704548.

[9] L. Qiao, S. Zhang, H. Xiao, D. Singh, K. Zhang, Z. Liu, et al., Orbital controlled band gap engineering of tetragonal BiFeO 3 for optoelectronic applications, Journal of Materials Chemistry C, 6(2018) 1239-47.

[10] Z. Xu, H. Cui, G. Zhang, Pd-decorated WTe2 monolayer as a favorable sensing material toward SF6 decomposed species: A DFT study, Acs Omega, 8(2023) 4244-50.
